# Targeting the glutamine metabolism to suppress cell proliferation in mesenchymal docetaxel-resistant prostate cancer

**DOI:** 10.1038/s41388-024-03059-4

**Published:** 2024-05-15

**Authors:** Alicia-Marie K. Beier, Celina Ebersbach, Tiziana Siciliano, Jana Scholze, Jörg Hofmann, Pia Hönscheid, Gustavo B. Baretton, Kevin Woods, Borhane Guezguez, Anna Dubrovska, Sascha D. Markowitsch, Christian Thomas, Martin Puhr, Holger H. H. Erb

**Affiliations:** 1 Department of Urology, Technische Universität Dresden, Dresden, Germany; 2 Institute of Pathology, Universitätsklinikum Carl Gustav Carus Dresden, 01307 Dresden, Germany; 3 National Center for Tumor Diseases (NCT), Partner Site Dresden, Dresden, Germany; 4 IIIrd Department of Medicine - Hematology & Oncology, University Medical Center of the Johannes Gutenberg-University, Mainz, Germany; 5 German Cancer Consortium (DKTK), Heidelberg, Germany; 6 OncoRay-National Center for Radiation Research in Oncology, Faculty of Medicine and University Hospital Carl Gustav Carus, Technische Universität Dresden and Helmholtz-Zentrum Dresden-Rossendorf, Dresden, Germany; 7 Helmholtz-Zentrum Dresden-Rossendorf, Institute of Radiooncology-OncoRay, Dresden, Germany; 8 National Center for Tumor Diseases (NCT), Dresden, Germany; German Cancer Research Center (DKFZ), Heidelberg, Germany; Faculty of Medicine, University Hospital Carl Gustav Carus, Technische Universität Dresden, Dresden, Germany; Helmholtz-Zentrum Dresden-Rossendorf (HZDR), Dresden, Germany; 9 German Cancer Consortium (DKTK), Partner Site Dresden and German Cancer Research Center (DKFZ), Dresden, Germany; 10 Department of Urology and Pediatric Urology, University Medical Center Mainz, Mainz, Germany; 11 Medical University of Innsbruck, Department of Urology, 6020 Innsbruck, Austria

**Keywords:** Cancer metabolism, Prostate cancer

## Abstract

Docetaxel (DX) serves as a palliative treatment option for metastatic prostate cancer (PCa). Despite initial remission, acquired DX resistance is inevitable. The mechanisms behind DX resistance have not yet been deciphered, but a mesenchymal phenotype is associated with DX resistance. Mesenchymal phenotypes have been linked to metabolic rewiring, obtaining most ATP production by oxidative phosphorylation (OXPHOS) powered substantially by glutamine (Gln). Likewise, Gln is known to play an essential role in modulating bioenergetic, redox homeostasis and autophagy. Herein, investigations of Gln deprivation on DX-sensitive and -resistant (DR) PCa cells revealed that the DR cell sub-lines were susceptible to Gln deprivation. Mechanistically, Gln deprivation reduced OXPHOS and ATP levels, causing a disturbance in cell cycle progression. Genetic and chemical inhibition of the Gln-metabolism key protein GLS1 could validate the Gln deprivation results, thereby representing a valid therapeutic target. Moreover, immunohistological investigation of GLS1 revealed a high-expressing GLS1 subgroup post-docetaxel failure, exhibiting low overall survival. This subgroup presents an intriguing opportunity for targeted therapy focusing on glutamine metabolism. Thus, these findings highlight a possible clinical rationale for the chemical inhibition of GLS1 as a therapeutic strategy to target mesenchymal DR PCa cells, thereby delaying accelerated tumour progression.

## Introduction

Docetaxel (DX) is a widely used chemotherapy drug in cancer treatment, including metastatic prostate cancer (PCa), the second most common cancer in men and the fifth leading cause of cancer-related death in men worldwide [1–4]. In castration-resistant prostate cancer (CRPC), docetaxel treatment significantly increased overall survival, reduced pain, decreased prostate-specific antigen serum levels, and enhanced quality of life. However, approximately 40–45% of patients with metastatic (m)CRPC do not respond to docetaxel (DX) or develop therapy resistance [5, 6]. Therefore, new therapeutic strategies are required to develop new and more effective treatments to improve docetaxel and treat docetaxel-resistant (DR) mCRPC.

Several mechanisms of docetaxel resistance have been identified, including increased drug efflux, drug-binding site mutations in microtubules, and increased anti-apoptotic and pro-survival pathways [7, 8]. Moreover, in vitro and immunohistological studies demonstrated that docetaxel-resistant mCRPC has an increased population of cells with a mesenchymal phenotype [9]. Moreover, patients with tumour relapse after docetaxel treatment have significantly reduced E-cadherin expression, indicating an elevated mesenchymal cell sub-population [9].

Mesenchymal cancer cells have already been linked to altered invasion and motility properties and seem to adopt a cancer stem cell (CSC)-like phenotype and consequently increased tumour-forming potential [9–11]. Due to these adaptations, distinct energy requirements are needed, resulting in the reprogramming of metabolism to prioritise energy production by increased oxidative phosphorylation (OXPHOS), glutathione (GSH) production, and reactive oxygen species (ROS) scavenging [12]. These features are associated with the glutamine (Gln) metabolism [13, 14].

The non-essential amino acid Gln is the second most common extracellular nutrient that fuels cancer cell metabolism to sustain cell growth and proliferation [13]. Due to their dependency on amino acids, these cancers become addicted to Gln. In primary PCa, lipids, succinate, and pyruvate are the primary energy sources. Therefore, Gln plays a lesser role at this tumour stage [14]. However, during disease progression to CRPC, Gln is an essential factor [14, 15]. Gln provides a source of carbon and nitrogen groups for the tricarboxylic acid (TCA) cycle to synthesise biomolecules such as ATP, NADH, nucleotides, proteins, and lipids [14]. To this end, glutaminase (GLS)1 and 2 transform Gln into glutamate, which is converted to α-ketoglutarate, a component utilised by the TCA [14]. In addition, Gln serves as a building block for the synthesis and regeneration of the antioxidant glutathione and as a co-factor for DNA and histone demethylases [14].

Several studies have investigated Gln metabolism as a possible therapeutic option in PCa. However, none of these studies has assessed Gln metabolism as a possible therapeutic target in DR cells with a mesenchymal phenotype. Therefore, this study investigated the influence of Gln deprivation during chemotherapeutic treatment in DX-sensitive and DR PCa cell models and determined its therapeutic value in chemotherapy-resistant cells with a mesenchymal phenotype.

## Results

### The proliferation of docetaxel-resistant cells is more dependent on glutamine depletion

**Fig. 1:**
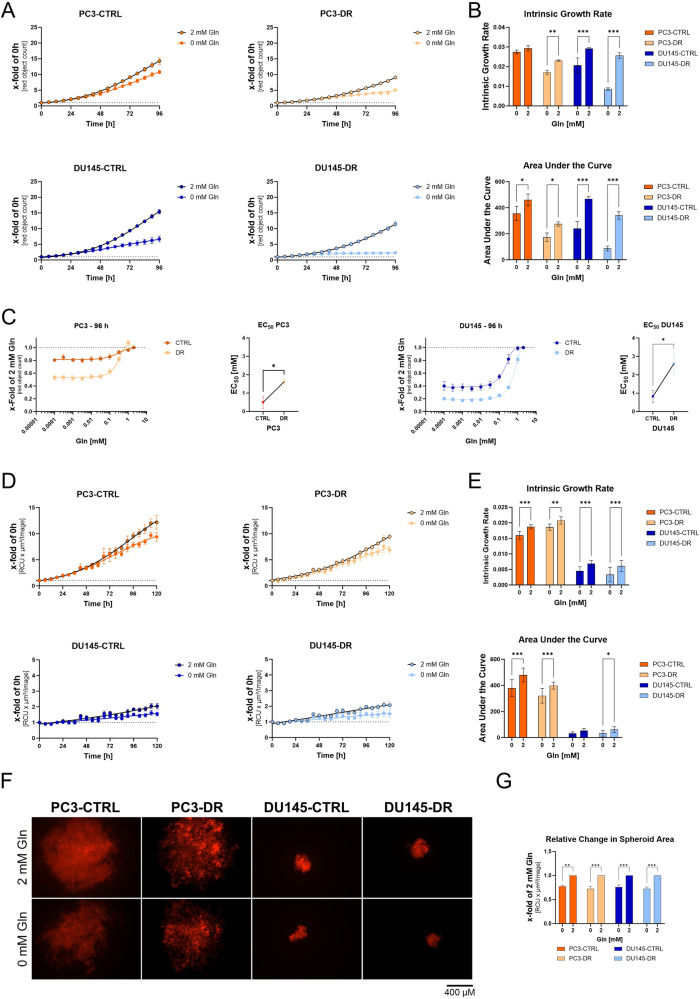
Proliferation of docetaxel-resistant PCa cells is highly dependent on Gln. **A** Influence of Gln deprivation on PC3-CTRL, PC3-DR, DU145-CTRL, and DU145-DR cell proliferation for 96 h. Curve fitting was performed using Prism. Values are expressed as mean ± SEM relative to 0 h. **B** Relative changes in intrinsic growth rates (IGR) and area under the curve (AUC) values were calculated from the growth curve experiments. **C** Dose-response curves of different concentrations of Gln and graphical illustration of the change in EC_50_ values of PC3-CTRL, PC3-DR, DU145-CTRL, and DU145-DR cell proliferation compared to 2 mM Gln. Data were plotted as mean ± SEM of the three biological replicates. Significant differences were identified using paired Student T-Test. **D** Influence of Gln deprivation on PC3-CTRL, PC3-DR, DU145-CTRL, and DU145-DR cell proliferation for 96 h. Curve fitting was performed using Prism. **E** Relative changes in IGR and AUC values were calculated from the spheroid growth curve experiments. **F** Representative spheroids pictures of PC3-CTRL, PC3-DR, DU145-CTRL, and DU145-DR after 96 h Gln deprivation (scale bar = 100 µm). **G** Relative change in the spheroid area compared to 2 mM Gln. Data were plotted as mean ± SEM of the three biological replicates. Significant differences were identified using One-way ANOVA. All differences highlighted by asterisks were statistically significant (*p ≤ 0.05. **p ≤ 0.01 ***p ≤ 0.001).

Gln is an essential nutrient for cancer cells, including PCa [13–15]. Therefore, the effect of Gln deprivation on DX-sensitive and DX-resistant PC3 and DU145 cells was evaluated (Fig. 1). The established cell lines showed a mKATE2 positivity of 90-100% after blasticidin selection (Supplementary Fig. 1A). Moreover, IncuCyte^®^ S3 Live Cell Analysis System analysis revealed that the increase in red object count was significantly correlated with the increase in confluence (Supplementary Fig. 1A). Only the red object count data are shown in this manuscript for readability reasons. To validate the docetaxel resistance of the selected cell lines, 2000 PC3 and DU145 cells/well were cultured in a 96-well plate and treated with different concentrations of DX (0.1 nM to 10 µM) for 96 h (Supplementary Fig. 1C). All tested DR cells demonstrated an at least 10-fold increase in IC_50_ values after DX treatment compared to CTRL cells, confirming DX resistance in DR cells. Initial studies show that 10% FBS has a significant reduction on the growth inhibition of glutamine deprivation (Supplementary Fig. 1D). Therefore, to reduce the possibility of compensatory effects of nutrients in FBS, FBS was reduced to 5% for all experiments, as the reduction had little effect on the proliferation of the used cell lines (Supplementary Fig. 1E).

To assess the influence of Gln on cell proliferation, cells were starved for 24 h and subsequently treated with different concentrations of Gln. Changes in proliferation were evaluated using the IGR and AUC. IGR and AUC revealed that all tested cell lines showed significantly diminished cell proliferation when cultured without Gln (Fig. 1A+B). Gln dose-response analysis after 96 h Gln deprivation (Fig. 1C) revealed increased sensitivity to Gln deprivation in the DX docetaxel-resistant PC3 and DU145 cells, as shown by the increased effective dose (EC)_50_ values in the docetaxel-resistant cells (Fig. 1C). In PC3, the EC_50_ increased from 0.5 mM to 1.6 mM in DR cells. In DU145, the EC_50_ increased from 0.8 mM to 2.6 mM in DR cells. To assess the influence of Gln deprivation on spheroid growth of the DX-sensitive and-resistant PC3 and DU145 cell lines, spheroids of the cell lines were starved for 24 h and subsequently cultured with or without 2 mM Gln. A comparison of IFR and AUC revealed that all spheroids showed reduced growth without Gln (Fig. 1D+E), resulting in a reduced spheroid area after 120 h (Fig. 1G). However, there was no difference in spheroid size between CTRL and DR cells.

### Influence of Gln deprivation on metastatic potential

Metastasis is a complex process involving migration, invasion, adhesion, and re-growth in the second organ’s microenvironment [16]. Therefore, clonogenic potential (CFE), migration, invasion, and adhesion potential were used to test the influence of Gln on metastatic potential.

**Fig. 2:**
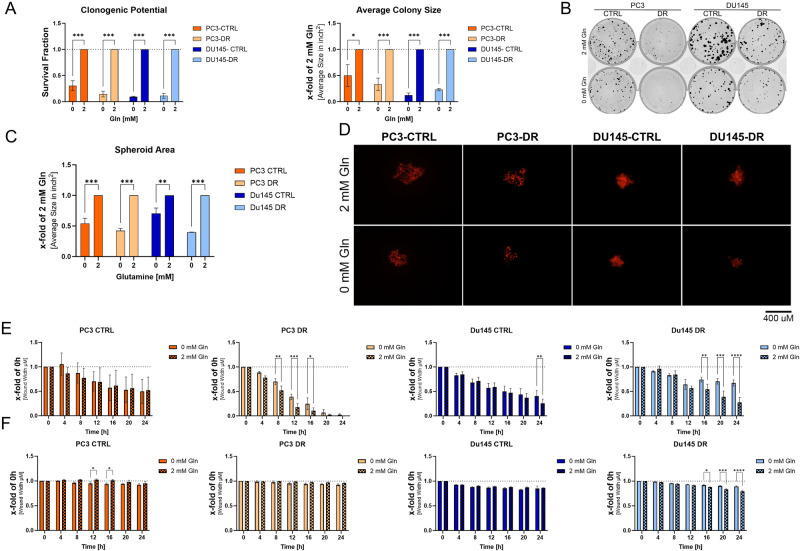
Clonogenic potential of PCa cells is reduced by Gln deprivation. **A** Clonogenic assays of PC3-CTRL, PC3-DR, DU145-CTRL, and DU145-DR cells after Gln withdrawal of PC3-CTRL, PC3-DR, DU145-CTRL, and DU145-DR. Survival Fraction represents the relative change in colony number (⩾50 cells/colony). Average colony size represents the relative change in colony area. Data are shown as relative changes compared to 2 mM Gln and was scored 10 days after plating. The results are plotted mean ± SEM of four biological replicates. Significant differences were identified using One-way ANOVA. **B** Representative images of the clonogenic assays. **C** Relative change in colony size of freshly formed colonies. Data are shown as relative changes compared to 2 mM Gln and was scored 4 days after plating. The results are plotted mean ± SEM of four biological replicates. **D** Representative images of the formed spheroids (scale bar = 100 µm). **E** Relative change of cell migration in PC3-CTRL, PC3-DR, DU145-CTRL, and DU145-DR cells after Gln deprivation. Data are expressed as relative change of wound width in μm and are the mean ± SEM of three independent experiments. **F** Relative change of cell invasion in PC3-CTRL, PC3-DR, DU145-CTRL, and DU145-DR cells after Gln deprivation. Data are expressed as relative change of wound width in μm and are the mean ± SEM of three independent experiments. Significant differences were identified using Two-way ANOVA. All differences highlighted by asterisks were statistically significant (*p ≤ 0.05. **p ≤ 0.01 ***p ≤ 0.001).

Reattachment of cells to different matrices is essential for metastasis. Therefore, the influence of Gln deprivation on the ability of cells to reattach to different matrices was tested. To this end, DX-sensitive and-resistant PC3 and DU145 cells were cultured for 96 h with or without 2 mM Gln, and adhesion assays were performed using no coating, normal associated fibroblasts, cancer-associated fibroblasts, human umbilical vein endothelial cells (HUVEC), Matrigel™, and poly-D-lysine. The adhesion assays revealed that all Gln-starved cell lines did not decrease in their adhesion compared to their controls (Supplementary Fig. 2A). Clonogenic assays revealed that all tested cell lines showed a significant decrease in survival fraction and average colony size below 0.1 mM Gln (Fig. 2A, Supplementary Fig. 2B+C). Consistent with this, Gln deprivation reduced spheroid formation in all cell lines (Fig. 2C+D). For scratch wound assays, PC3 and DU145 cells were seeded and Gln starved for 24 h, and wound width was assessed with and without Gln for 48 h (Fig. 2E+F, Supplementary Fig. 2E+F). PC3 and DU145 CTRL cells showed negligible effects on cell migration without Gln (Fig. 2E, Supplementary Fig. 2E). In contrast, PC3-DR cells show a significant decrease in migration after 4 h and DU145-DR after 16 h. Assessment of the cell invasion revealed only a significant change in cell invasion in DU145-DR cells after 16 h (Fig. 2F, Supplementary Fig. 2D). It can therefore be deduced from the migration and invasion attempts that Gln plays only a negligible role in these mechanisms.

### Gln deprivation reduces mitochondrial functions and induces apoptosis in docetaxel-resistant cells

**Fig. 3:**
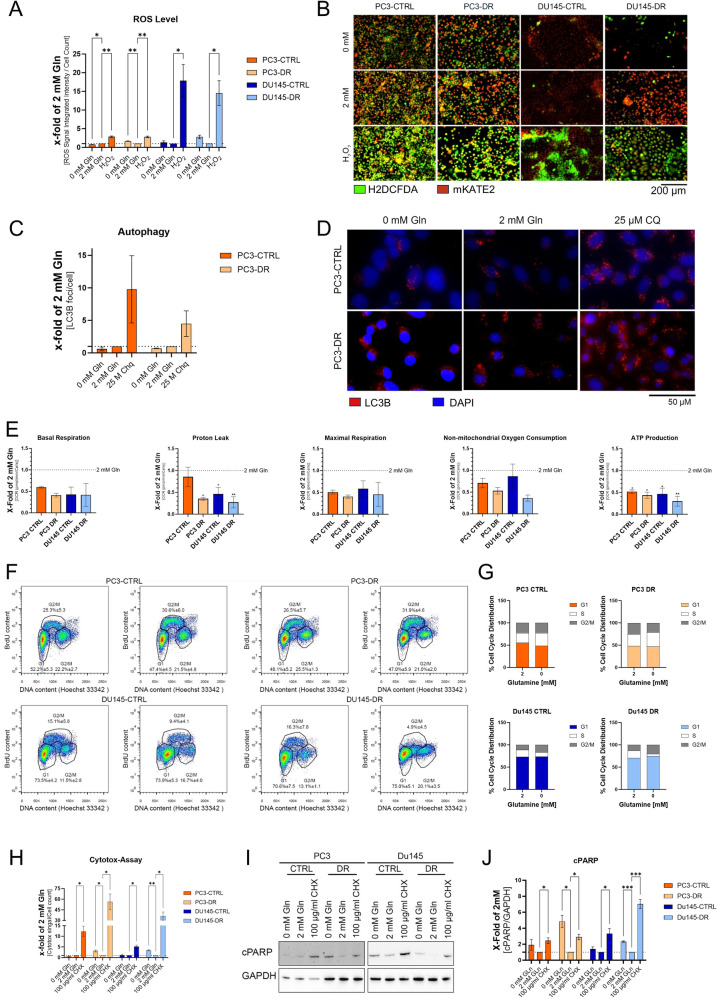
Gln deprivation reduces ATP production and changes in cell cycle phases. **A** Relative ROS level changes were detected with the ROS indicator 2′,7′-dichlorodihydrofluorescein diacetate (H2DCFDA) after 96 h of Gln deprivation. ROS indicator was normalised to cell number and the results are plotted as mean ± SEM of five biological replicates. Significant differences were identified using One-way ANOVA. **B** Representative images of the immunofluorescence pictures for ROS analysis (scale bar = 200 µm). **C** Relative change of LC3-positive puncta (autophagosomes) of PC3-CTRL and PC3-DR cells starved for Gln for 96 h. 25 µM chloroquine (CQ) treated cells were used as a positive control. The autophagosome number was normalised to the cell number. The results are plotted mean ± SEM of five biological replicates. **D** Representative images of the immunofluorescence pictures for ROS analysis (scale bar = 50 µm). **E** Relative mitochondrial respiration changes with basal respiration, proton leak, maximal respiration, non-mitochondrial oxygen consumption, and ATP production. Data were obtained by performing a Seahorse Cell Mito Stress analysis. The results are plotted mean ± SEM of three biological replicates. Significant differences were identified using one-way ANOVA. **F** Representative cytograms of cell cycle analysis after 96 h Gln deprivation using the Click-iT™ Plus EdU 488 Flow Cytometry Assay Kit. **G** Distribution of cell cycle phases after 96 h Gln deprivation. **H** Relative changes in cell death using the IncuCyte® Cytotox assay after 96 h Gln deprivation. As a positive control, cells were treated with 25 µg/ml Cycloheximide (CHX). The Cytotox signal was normalised to cell number and the results are plotted mean ± SEM of three biological replicates. Significant differences were identified using one-way ANOVA. **I** Representative Western Blot for cPARP after 96 h Gln deprivation. **J** Densiometric analysis of cPARP western blots normalised to GAPDH. Values are expressed as mean ± SD. Significant differences were identified using one-way ANOVA. All differences highlighted by asterisks were statistically significant (*p ≤ 0.05. **p ≤ 0.01 ***p ≤ 0.001).

Gln deprivation has been associated with changes in ROS levels, autophagy, cell cycle arrest, and apoptosis [17]. To this end, the cell lines were starved for 24 h, followed by a 96 h incubation with and without Gln. Subsequently, ROS levels, autophagy, mitochondrial function, the cell cycle, and apoptosis were assessed. Gln deprivation resulted in no change in ROS levels in the tested cell lines (Fig. 3A+B). This result is supported by rescue experiments with reduced GSH treatment, which showed no rescuing effect on cell proliferation after Gln deprivation (Supplementary Fig. 3A). In addition, the autophagy level did not change after 96 h of Gln deprivation compared with the controls in the tested cell lines (Fig. 3C+D). In addition, cell mitochondrial stress assays were performed to assess changes in mitochondrial function after 96 h of Gln deprivation. Seahorse analysis revealed a reduced oxygen consumption rate (OCR, Supplementary Fig. 4B), significantly reducing ATP production in all tested cell lines (Fig. 3E). Gln has also been reported to regulate cell-cycle progression [18]. Cell cycle analysis revealed increased S-phase in PC3 CTRL and DR cells after Gln withdrawal and increased G2/M Phase after Gln withdrawal (Fig. 3F+G, Supplementary Fig. 3C). Treatment with 2 mM Gln of the DU145 cells after 96 h Gln deprivation resulted in the induction of cell proliferation (Supplementary Fig. 3D). To assess the general viability changes after Gln deprivation, cytotoxicity assays revealed a significant increase in cytotoxicity after 96 h of Gln deprivation in DR cells (Fig. 3H). This increase in cytotoxicity was accompanied by a significant increase in the apoptosis marker cPARP in DR cells (Fig. 3I+J).

### Influence of Gln on DX treatment efficiency

To assess if Gln deprivation influences DX treatment efficiency, 2000 PC3 and DU145 cells/well were seeded in a 96-well plate, Gln starved for 24 h and subsequently treated with different DX concentrations (0.1 nM to 10 µM) combined with different Gln concentrations (0–2 mM) for 96 h (Supplementary Fig. 2A). The dose-response curve of PC3-CTRL and DU145-CTRL revealed that with lower Gln, IC_50_ values increased for DX treatment (Supplementary Fig. 2A). The calculated CI values indicated DX antagonism by Gln deprivation (Fig. 2B). The PC3-DR and DU145-DR cell dose–response curves revealed no decrease in IC_50,_ indicating that Gln did not influence DR (Supplementary Fig. 2C).

### GLS1 expression is elevated in PCa tissue

**Fig. 4:**
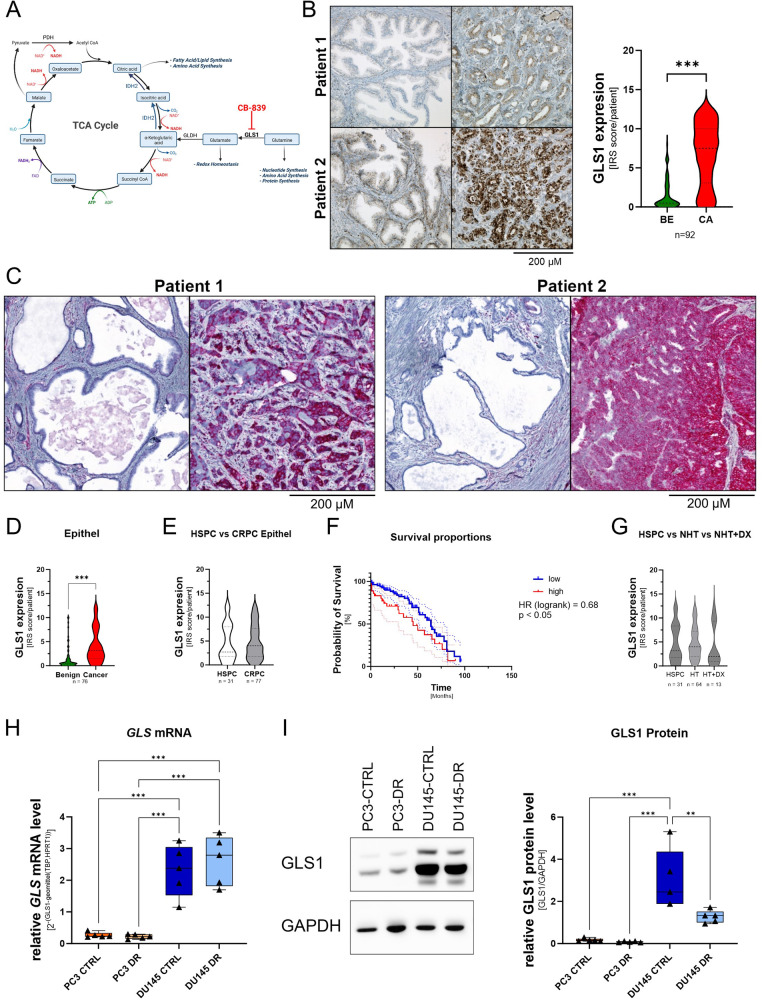
GLS1 is elevated in PCa. **A** Schematic overview of GLS1 role in the Gln metabolism in cancer. Created with BioRender.com. **B** Representative microscopy images and the quantification of GLS1 expression in benign and primary PCa tissues of the Innsbruck cohort. GLS1 expression was quantified using the immune-reactivity scores (IRS) of paired benign and malignant prostate areas of 92 patients (scale bar = 200 µm). Data are plotted as a violine blot. Significant differences were identified using one-way ANOVA. **C** Representative microscopy images of GLS1 expression in the Dresden cohort’s benign and primary PCa tissues (scale bar = 200 µm). **D** Quantifying the GLS1 expression of the Dresden cohort using the immune-reactivity scores (IRS) of paired benign and malignant prostate areas of 76 patients. Data are plotted as a violine blot. Significant differences were identified using one-way ANOVA. **E** Quantifying the GLS1 expression in HPSC (*n* = 31) and CRPC patients (*n* = 77) of the Dresden cohort using the immune-reactivity scores (IRS). Data are plotted as a violine blot. **F** Kaplan–Meier curves indicating OS according to the GLS1 expression level of the Dresden cohort. The median GLS1-IRS was chosen as the threshold. **G** Quantifying the GLS1 expression in the HSPC (*n* = 31), hormone treatment (HT, *n* = 64, including LHRH agonist, abiraterone, or enzalutamide-treated patients), and hormone treatment and docetaxel (HT + DX, *n* = 13) sub-groups. Data are plotted as a violine blot. **H** *GLS* mRNA levels in PC3-CTRL, PC3-DR, DU145-CTRL, and DU145-DR cells normalised to the geometric mean of TBP and HPRT1. Values are expressed as box and whisker plots (min to max). Significant differences were identified using One-way ANOVA. **I** Representative Western Blot and densitometric analysis for GLS1 normalised to GAPDH in PC3-CTRL, PC3-DR, DU145-CTRL, and DU145-DR cells. Values are expressed as box and whisker plots (min to max). Significant differences were identified using one-way ANOVA. All differences highlighted by asterisks were statistically significant (**p ≤ 0.01 ***p ≤ 0.001).

GLS1 plays a crucial role in glutaminolysis (Fig. 4A) and several studies have reported tumour-reducing effects of GLS1 inhibition by CB-839 [19–24]. To assess GLS1 expression in PCa, GLS1 expression was analysed in TMAs of two independent cohorts, the Innsbruck and Dresden cohorts, by immunohistochemistry (IHC). Antibody specificity was assessed using the controls specified by the antibodies datasheet and with PC3 cells transfected with siRNA specific for GLS1 (Supplementary Fig. 4D+E). The Innsbruck cohort reveals elevated GLS1 expression in cancerous areas compared to the corresponding benign areas (Fig. 4B). However, GLS1 expression did not change with the Gleason score or T stage (Supplementary Fig. 5A). In line, the Dresden cohort also showed elevated GLS1 expression in cancerous areas (Fig. 4C), which did not change with hormone status (Fig. 4E). Kaplan–Meier analysis revealed a significant reduction in the median overall survival (OS, Hazard Ratio log-rank = 0.68) for patients with high GLS1 from 65 to 45 months (Fig. 4F). To assess whether GLS1 expression in patients was influenced by treatment, the Dresden cohort was subdivided into treatment-naïve, hormone-treated (HT), and HT combined with docetaxel (HT + DX, Fig. 4G). Compared to the treatment-naïve cohort, there was no change in median GLS1 expression (Fig. 4G). However, GLS1 expression seemed to populate into high and low GLS1 expression after hormone treatment (HT) combined with docetaxel (HT + DX, Fig. 4G, Supplementary Fig. 5B). In the HT + DX cohort, Kaplan–Meier analysis revealed a significant reduction in median overall survival (OS, Hazard Ratio log-rank = 0.13) for patients with high GLS1 from 51 to 6 months (Supplementary Fig. 5C).

To analyse GLS1 expression in the selected cell lines, qPCR and western blot analyses were performed (Fig. 4H+I, Supplementary Fig. 5D). mRNA (Fig. 4H) and protein (Fig. 4I) expression levels were significantly higher in the DU145 cell lines than in the PC3 cell lines.

### Influence of siGLS1 on cell proliferation

**Fig. 5:**
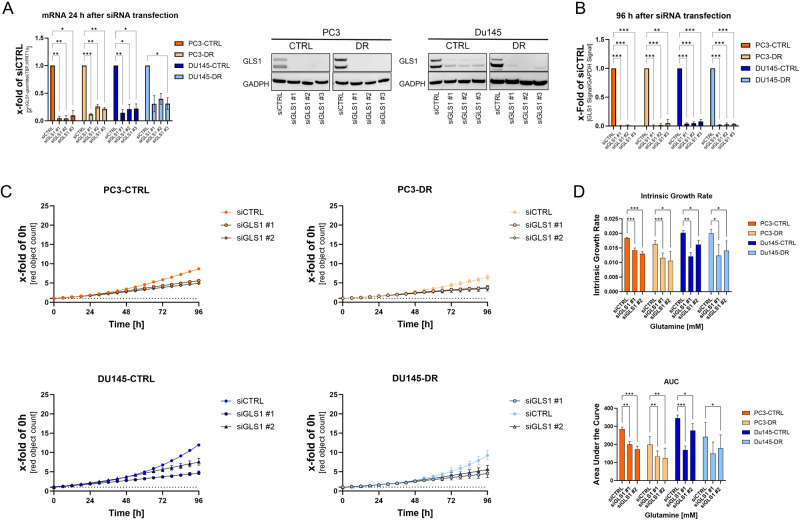
GLS1 knockdown reduces cell proliferation in PCa cell lines. **A** Relative change of *GLS* mRNA levels after 24 h siRNA transfection in PC3-CTRL, PC3-DR, DU145-CTRL, and DU145-DR cells normalised to the geometric mean of TBP and HPRT1. Values are expressed mean ± SEM. Significant differences were identified using one-way ANOVA. **B** Representative Western Blot and densitometric analysis for changes in GLS1 protein levels after 96 h siRNA transfection in PC3-CTRL, PC3-DR, DU145-CTRL, and DU145-DR cells normalised to GAPDH. Values are expressed mean ± SD. Significant differences were identified using one-way ANOVA. **C** Influence of GLS1 knockdown on PC3-CTRL, PC3-DR, DU145-CTRL, and DU145-DR cell proliferation for 96 h. Curve fitting was performed using Prism. Values are expressed as mean ± SEM relative to 0 h. **D** Relative changes in intrinsic growth rates and area under the curve values were calculated from the growth curve experiments. All differences highlighted by asterisks were statistically significant (*p ≤ 0.05. **p ≤ 0.01 ***p ≤ 0.001).

A siRNA approach using three different siRNAs was used to assess the impact of GLS1 on the proliferation of DX-sensitive and DX-resistant PC3 and DU145 cells. Specific GLS1 knockdown (siGLS#1-3) resulted in significantly reduced GLS1 mRNA expression after 24 h (Fig. 5A). Consistent with this, GLS1 downregulation was observed 24 h after transfection and was stable for at least 96 h, representing the experimental time window (Fig. 5B, Supplementary Fig. 6). As all tested siRNAs showed similar efficiency, siGLS#1 and siGLS#2 were used for subsequent experiments. To assess the effect of siGLS on proliferation, cells were transfected 24 h after seeding in a medium without Gln and incubated for 96 h with a medium containing 2 mM Gln. Compared to the scrambled control (siCTRL), siGLS-transfected cells showed significantly diminished IGE and AUC (Fig. 5C).

### CB-839 reduces cell proliferation and clonogenic potential of DX-sensitive and resistant PCa cell lines

**Fig. 6:**
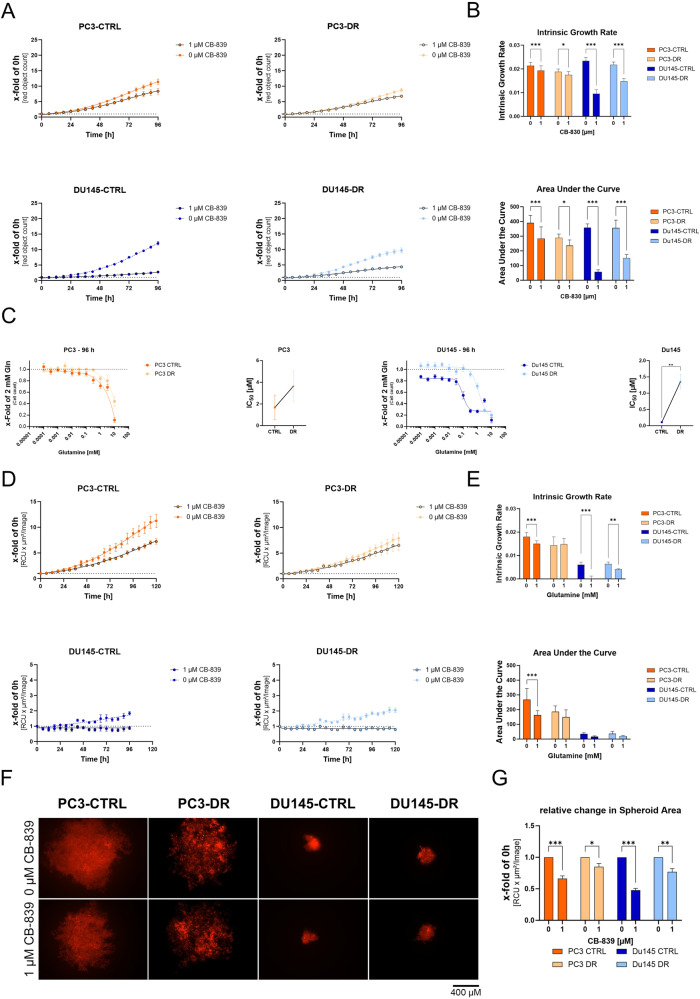
Chemical inhibition of Gln metabolism by CB-839 reduces the proliferation of PCa cells. **A** Influence of 1 µM CB-839 on PC3-CTRL, PC3-DR, DU145-CTRL, and DU145-DR cell proliferation for 96 h. Curve fitting was performed using Prism. Values are expressed as mean ± SEM relative to 0 h. **B** Relative changes in intrinsic growth rates and area under the curve values were calculated from the growth curve experiments. Significant differences were identified using One-way ANOVA. **C** Dose–response curves of different concentrations CB-839 and graphical illustration of the change in IC_50_ values of PC3-CTRL, PC3-DR, DU145-CTRL, and DU145-DR cell proliferation compared to 2 mM Gln. Data were plotted as mean ± SEM of the three biological replicates. Significant differences were identified using paired Student *T*-Test. **D** Influence of CB-389 on PC3-CTRL, PC3-DR, DU145-CTRL, and DU145-DR cell proliferation for 96 h. Curve fitting was performed using Prism. **E** Relative changes in intrinsic growth rates and area under the curve values were calculated from the spheroid growth curve experiments. Data were plotted as mean ± SEM of the three biological replicates. **F** Representative spheroids pictures of PC3-CTRL, PC3-DR, DU145-CTRL, and DU145-DR after 96 h treatment with CB-839 (scale bar = 100 µm). **G** Relative change in the spheroid area after treatment with 1 µM CB-839. Data were plotted as mean ± SEM of the three biological replicates. Significant differences were identified using One-way ANOVA. All differences highlighted by asterisks were statistically significant (*p ≤ 0.05. **p ≤ 0.01 ***p ≤ 0.001).

To assess the influence of the GLS1 inhibitor CB-839 on the proliferation of DX-sensitive and -resistant cells, cells were starved for 24 h and subsequently treated with a medium containing 2 mM Gln and different concentrations of CB-839 (0–10 µM). Treatment with 1 µM CB-839 resulted in significantly diminished IGE and AUC (Fig. 6A+B). CB-839 dose-response analysis after 96 h (Fig. 6C) revealed increased sensitivity to CB-839 in DX-resistant PC3 and DU145 cells, as shown by the increased IC_50_ values in the DX-resistant cells (Fig. 6C). High GLS1-expressing DU145 cells generally showed a higher sensitivity to CB-839 than low GLS1-expressing PC3 cells. A similar result was observed in the change in survival fraction (Supplementary Fig. 7A) and spheroid growth (Fig. 6D+E), showing a lower efficiency of CB-839 in DR cells. Migration and invasion after treatment with CB-839 align with the results reported for Gln deprivation (Supplementary Fig. 2C+D, Supplementary Fig. 7B+C). Seahorse analysis also revealed a less effective reduction in OCR after CB-839 treatment (Supplementary Fig. 7D), resulting in a less significant reduction in ATP production in DR cells than in CTRL cells (Supplementary Fig. 7E). Previous studies have revealed that DR cells express the ABCB1 transporter, leading to multidrug resistance in multiple cancer types, including PCa [8, 25]. To assess whether the ABCB1 transporter is responsible for the lower sensitivity of DR cells to CB-839, DR cells were treated with 1 µM CB-839 with or without 50 nM of the ABCB1 transporter inhibitor elacridar (Supplementary Fig. 7F). Treatment with elacridar increases the sensitivity to CB-839 in the DR cells, as indicated by the decrease in IC_50_ values. These results confirm the possibility of reducing DR cell proliferation by chemical GLS1 inhibition. However, ABCB1 transporter efflux activity may mediate resistance to the GLS1 inhibitors.

## Discussion

Metabolic rewiring towards Gln metabolism is a well-established mechanism to fuel the energetic needs of cancer cells and CRPC [14]. Gln drives energy production and is an essential nitrogen and carbon donor for the biosynthesis of amino acids, nucleotides, and fatty acids [14]. However, the involvement of Gln metabolism in docetaxel-resistant CRPC cells with mesenchymal phenotype has not yet been investigated. Cancer cells with mesenchymal phenotypes have been linked to therapy resistance and stem cell features in multiple cancers, including PCa [9, 26–29]. Ippolito and colleagues demonstrated that docetaxel-resistant PC3 and DU145 cells shift their metabolism to OXPHOS and that the metabolic adaptation is related to the mesenchymal cell phenotype, which has been associated with increased features of the Gln metabolism [14, 30]. Proliferation analysis of DX-sensitive and -resistant CRPC cells performed in this study revealed a more substantial decrease in growth rate in DX-resistant cells after Gln deprivation. In line, Gln dose-response experiments revealed an increased sensitivity to Gln deprivation of DX-resistant cells. These results were similar to previous observations in breast cancer and soft tissue sarcoma growth, demonstrating increased sensitivity to Gln deprivation in cells with a mesenchymal-like phenotype [31, 32].

Metastasis is associated with advanced PCa staging and poor prognosis [33]. Increased Gln utilisation of PCa cells has been linked to increased metastatic potential [34, 35]. Moreover, inhibition of Gln uptake decreased tumour growth and metastasis [36]. Glutaminolysis inhibition has generally reduced disease recurrence risk and metastases in several tumour entities [35, 37]. Therefore, the influence of Gln on the clonogenic potential, migration, invasion, and adhesion was investigated within this study. All tested cell lines showed little clonogenic potential and reduced colony size without Gln. These results align with studies from different tumour entities, demonstrating reduced clonogenic potential in the absence of Gln or following inhibition of its uptake [38–41]. In contrast to the proliferation data, the DX-resistant cells’ clonogenic potential was not significantly reduced. However, the clonogenic potential of the DX-sensitive cells has already been strongly diminished. Consequently, the colony-forming ability of the chosen CRPC cells seems to be highly dependent on Gln. The inhibitory influence of Gln deprivation on the clonogenic potential is strengthened by the fact that adherence does not change after Gln deprivation, indicating the same cell numbers at the beginning of the experiments. Besides the clonogenic potential, the influence of Gln on migration and invasion of DX-sensitive and -resistant cells was investigated. Prasad and colleagues reported reduced migration and invasion of ovarian cancer cells after Gln deprivation [42]. The study suggested that Gln deprivation results in the deactivation of the transcription factor ETS1, which is responsible for the expression of vimentin and metalloproteases, proteins involved in cancer cell migration and invasion [42, 43]. The results obtained in this study indicate that only the migration of DX-resistant cells is reduced by Gln withdrawal, whereas invasion is neglectable influenced. These results suggest that Gln plays an essential role in the proliferation and parts of the metastatic process of DX-sensitive and -resistant mCRPC cells. However, DX-resistant cells seem more dependent on Gln in these processes.

Due to Gln’s multifaceted role in various cellular processes, Gln deprivation has been associated with apoptosis, autophagy, and cell-cycle arrest [17]. Previous studies by Mukah et al. revealed inhibition of autophagy after Gln-deprivation in the hormone-sensitive LNCaP cell line. In contrast, Gln-deprivation increased intracellular ROS levels and induced apoptosis in the mCRPC cell line DU145 [19]. In contrast to these results, Gln deprivation did not affect autophagy or ROS levels of the tested DX-sensitive and -resistant mCRPC cells. However, analysis of OCR after Gln-deprivation indicates a reduction in mitochondrial OXPHOS. This reduction significantly decreases ATP levels, causing a cell cycle arrest. Similar effects have been reported by Gaglio and colleagues, who reported that Gln-deprivation severely slows down cell phase transit and, therefore, slows down cell proliferation [18]. Moreover, apoptosis induction could be observed in the DX-resistant mCRPC cells after Gln deprivation, partially explaining the higher sensitivity to Gln withdrawal.

An increase in OXPHOS has been described as a central feature of mediating docetaxel resistance [30]. Moreover, several mechanisms linked to DR, such as increased ATP-dependent efflux pump activity and Bcl-2 expression, have been linked to Gln metabolism [7, 44–46]. However, Gln withdrawal reduced the efficiency of DX treatment of DX-sensitive cells. This effect might result from the decreased growth rate caused by glutamine deprivation, which weakens the anti-cell division effect of DX. On the other hand, no resensitisation of DR cells to DX could be achieved by combination treatment with DX and Gln deprivation. These results indicate that DR is independent of the Gln metabolism.

The data from this study suggest that Gln metabolism is a potential therapeutic target in DR mCRPC. However, since reducing the amino acid in the body is impossible, various strategies have already been developed to target Gln-metabolism at multiple points [13]. Several studies have shown promising results by targeting GLS1, an essential protein in the Gln-metabolism in cancer cells [13, 14]. In particular, the GLS1 inhibitor CB-839 is an effective and selective GLS1 inhibitor, which has already demonstrated good tolerability in various phase I trials [47–49]. The inhibitor has also already shown promising results in several preclinical studies in PCa [19, 20, 50]. In line with reports of other tumour entities, immunohistochemistry analysis revealed a significant increase in GLS1 expression in malignant prostate tissue compared to benign areas. In addition, elevated GLS1 expression was associated with shorter OS [51]. The expression was independent of GS, TMN Stage, or HT treatment. This independence may result in the transcriptional regulation of GLS1 expression by MYC, a family of transcription factors that is also independently expressed by GS and TMN Stage [52].

However, specimens that failed treatment with HT + DX can be divided into high and low GLS1 expression sub-groups. Kaplan-Meier survival analysis of these patients reveals that high GLS1 expression is associated with shorter OS. As GLS1 is an essential part of the Gln metabolism, the different GLS1 expression groups indicate that DX treatment leads to DX-resistant PCa sub-groups. Those groups have a low and high dependency on Gln, of which the high GLS1 expressing group is associated with a bad prognosis. This result is in concordance with a previous study that identified GLS1 expression levels as a biomarker of PCa aggressiveness. However, the value of GLS1 as a prognostic therapy biomarker needs to be investigated in greater cohorts [19].

GLS1 has been reported to be a promising therapeutic target in PCa [19, 20, 50, 53]. This study could validate these observations by reducing cell proliferation using GLS1 knockdown and pharmacological inhibition. Dose-response experiments showed that cells with higher GLS1 expression responded better to the GLS1 inhibitor CB-839 than cells with lower GLS1 expression. This result strengthens the hypothesis that PCa cells with high GLS1 expression depend more on Gln metabolism. While cells with elevated GLS1 expression show enhanced responsiveness to CB-839, the opposite was observed in DR cells compared to their DS control cells. This conflicting outcome contradicts the heightened sensitivity of DR cells to Gln deprivation. It suggests that DR cells may metabolise Gln in a GLS1-independent way or have already developed a resistance mechanism to CB-839.

ATP-binding cassette (ABC) transporters efflux numerous structurally and biochemically unrelated compounds, thereby playing a central role in mediating multidrug resistance [54]. Moreover, they have been reported to play a crucial role in DR cell models used in this study [8]. As chemical inhibition of ABCB1 activity results in an increased sensitivity to CB-839 of the DR cells, there is evidence that these cells harbour a de novo CB-839 insensitivity. CB-839 is thus one of the drugs whose efflux activity is reduced by the efflux activity of ABCB1.

In conclusion, this study indicates that the Gln metabolism key protein GLS1 is a potential target in DR mCRPC cells with a mesenchymal phenotype and a biomarker of CRPC aggressiveness. Evidently, targeting the Gln metabolism reduces ATP production, causing a reduction in cell proliferation. In addition, DR cells seem more sensitive to Gln withdrawal but are de novo resistant to the already clinically tested GLS1 inhibitor CB-839. Future research is needed to develop novel GLS1 inhibitors to overcome already identified resistant mechanisms. Moreover, GLS1 expression levels appear to play an essential role in the efficacy of the CB-839 inhibitor. Therefore, clinical trials that have already been performed need to be re-analysed, including GLS1 expression data, and current studies must include GLS1 expression data to ascertain the potential therapeutic utility of GLS1 inhibitors.

## Materials and methods

### Chemicals

The following chemicals were used with concentrations as indicated in the results section and figure legends: dimethyl sulfoxide (DMSO, Cat# D2650, Sigma Aldrich, Merck KGaA Darmstadt, German), elacridar (Cat# S7772, Selleck Chemicals, Munich, Germany) L-glutathione reduced (GSH, Cat# G6013, Sigma Aldrich), L-glutamine (Gln, Cat# G8540, Sigma Aldrich), polybrene Infection/Transfection Reagent (Cat# TR-1003-G, Sigma Aldrich), docetaxel (DX, Cat# S1148, Selleck Chemicals), CB-839 (Cat# S7655, Selleck Chemicals), and blasticidin (Cat# ant-bl-05, InvivoGen SAS, Toulouse, France).

### Cell lines

The 293T cell line was obtained from the American Type Culture Collection (ATCC, Manassas, VA, USA). The DX-sensitive and DX-resistant PC3 and DU145 cell lines were kindly provided by Prof. Culig (Medical University of Innsbruck, Innsbruck, Austria) [9]. Cells were cultured under standard conditions (37 °C, humidified atmosphere with 5% CO_2_) in RPMI-1640 medium (Cat# 52400-025, Thermo Fisher Scientific, Frankfurt, Germany) supplemented with 10% fetal bovine serum (FBS; Cat# A5256701, Thermo Fisher Scientific). DX-resistant (DR) cell line medium was supplemented with 10 nM DX. Mycoplasma testing was performed according to the manufacturer’s instructions using a Mycoalert^®^ Detection Assay (Cat# LT07-318, Lonza, Basel, Switzerland). STR profiling was used to verify the cell line authentication. The characteristics of the cell lines are listed in Supplementary Table S1.

### Proliferation assay with the IncuCyte® S3 live cell analysis system

Cell proliferation was measured by mKATE2 labelled nuclei counting and confluence determination using the IncuCyte^®^ S3 Live-Cell Imaging System (Sartorius AG, Goettingen, Germany). The cells were seeded in 96-well clear flat-bottom plates (Cat# 3596, Corning GmbH, Kaiserslautern, Germany) and incubated overnight at 37 °C with 5% CO_2_. Subsequently, the plates were treated and placed into the IncuCyte^®^ S3 Live-Cell Imaging System live imaging system and scanned every 6 h. Confluence and cell number were analysed using IncuCyte 2023 C analysis software (Sartorius AG) by measuring the growth area or counting the mKATE2 labelled nuclei. Cell proliferation was expressed as increased cell confluence or number compared to the first scan time point or as an x-fold of untreated controls (CTRL).

### Patients material and immunohistochemistry (IHC)

Patients were selected from the Innsbruck PCa Biobank and the Tumour and Normal Tissue Bank of the University Cancer Center Dresden. The archived material was used according to the principles of the Declaration of Helsinki. It was approved by the Medical University of Innsbruck’s Ethics Committee (Study no. AN 1072/2018) and the Medical University of Dresden (Study no. EK59032007). Written consent was obtained from all patients and documented in the University Hospital Innsbruck (Austria) database and the medical hospital Carl Gustav Carus Dresden (Germany) in agreement with the statutory provisions. The Innsbruck cohort is represented using a tissue micro-array containing benign and primary cancer tissue cores from 120 treatment-naïve PCa patients who underwent open retropubic or robotic-assisted radical prostatectomy [55]. This cohort included 92 cancer tissue cores paired with benign tissue cores. The Dresden cohort contained 108 tissue specimens from PCa patients undergoing palliative TURP [56, 57]. Matched benign samples were excised from histologically confirmed nonmalignant regions of 76 patients. The baseline characteristics of the Dresden cohort patients are listed in Table 1. GLS1 IHC was performed using the Ventana BenchMark device (Roche, Vienna, Austria). The following antibodies were used: GLS1 (E9H6H) RabMab XP^®^ (1:800; Cat# 56750, Cell Signaling Technology, Frankfurt, Germany). The evaluation was performed using the following modified “quick-score” protocol: staining intensity was scored 0–4 (0 = absent, 1 = weak, 2 = intermediate, 3 = strong). The percentage of positively stained cells was scored 0–4 (0 = absent, 1 ≤ 10%, 2 ≤ 50%, 3 ≤ 75%, and 4 ≥ 75%). Both scores were multiplied to obtain an immunoreactivity score (IRS), ranging from 0 to 12 [55, 58].

**Table 1:** Baseline characteristics of the Dresden cohort patients.

	All	HSPC	CRPC	HT	HT + DX
Patients, n	108	31	77	64	13
Median age, years	73.5 ± 8.6	75.0 ± 7.7	73.0 ± 8.8	73.0 ± 9.0	66.0 ± 7.7
Overall survival, month	30.0 ± 25.6	48.0 ± 25.2	27.0 ± 23.8	73.5 ± 8.6	73.5 ± 8.6
Prior hormon treatment (LHRH agonist, abiraterone, or enzalutamide), %	59.3	0.0	83.1	100.0	100.0
Prior use of hormon treatment and docetaxel, %	12.0	0.0	16.9	0.0	100.0
Presence of metastases, %	39.8	6.5	57.1	52.3	100.0

*CRPC* Castration-resistant prostate cancer, *HSPC* Hormone-sensitive prostate cancer, *HT* Hormone treatment (LHRH agonist, abiraterone, or enzalutamide), *HT* *+* *DX* Hormone treatment (LHRH agonist, abiraterone, or enzalutamide) plus docetaxel

### Immunofluorescence

For immunofluorescence, 1250 cells were seeded into 8-well chamber slides and allowed to attach for 24 h. Subsequently, the cells were starved for 24 h, followed by treatment with medium with or without 2 mM Gln. After 96 h of incubation, the cells were washed with PBS and fixed with ice-cold methanol for 10 min, followed by a 1 h blocking step with PBS (Thermo Fisher Scientific) supplemented with 5% FBS (Thermo Fisher Scientific) and 0.3% Triton™ X-100 (Cat# X100-5ml, Sigma-Aldrich). For immunostaining, cells were incubated overnight with LC3B (D11) XP^®^ rabbit mAb (LOT:4, Cat# 3868, Cell Signaling Technology) diluted in antibody buffer containing PBS supplemented with 1% BSA (Cat#. 11930.04, SERVA Electrophoresis GmbH, Heidelberg, Germany) and 0.3% Triton X-100. After primary antibody incubation, cells were washed three times for 10 min with the antibody buffer, followed by 1 h incubation with the fluorescence-labelled F(ab′)2-Goat anti-Rabbit IgG (H + L) Secondary Antibody, Alexa Fluor™ 568 (Cat# A-11011, Thermo Fisher Scientific). Finally, the cells were washed three times with TBS, mounted with Vectashield Hard Set mounting medium with DAPI (Cat. H-1200-10, Vector Laboratories, Burlingame, USA), and covered with Precision cover glasses thickness No. 1.5H (Cat# 0107242, VWR International GmbH, Darmstadt, Germany). The cells were visualised using a Compact Fluorescence Microscope BZ-X800E (Keyence, Osaka, Japan) and analysed using BZ-X800 analysis software (Keyence, Osaka, Japan).

### Statistics

For all statistical analyses, such as curve fitting, area under the curve (AUC), statistical tests, and plotting, GraphPad Prism 10.0.3 (GraphPad Software, San Diego, CA, USA) was used. Data are presented as the mean ± SEM to estimate the various means in multiple repeated experiments. Unless otherwise noted, all experiments were performed with at least three biological replicates. The intrinsic growth rate (IGR) was calculated using ${\rm{r}}=[\frac{\mathrm{ln}\left(\frac{{\rm{N}}\left({\rm{t}}2\right)}{N(t1)}\right)}{({\rm{t}}2-{\rm{t}}1)}]$ [59]. The (CI) was calculated using the following equation: ${\rm{CI}}=[\frac{({\rm{C}}{\rm{A}},{\rm{X}}/{\rm{IC}}{\rm{X}},{\rm{A}})}{({\rm{C}}{\rm{B}},{\rm{X}}/{\rm{IC}}{\rm{X}},{\rm{B}})\,}]$ [60]. The Gaussian distribution was determined using the Kolmogorov-Smirnov and D’Agostino & Pearson omnibus normality tests. Student’s t-tests (two-sided) and one-way and two-way analyses of variance (ANOVA) were used to identify significant differences. Statistical significance was set at *p* ≤ 0.05. All differences highlighted by asterisks are statistically significant, as encoded in the figure legends (*p ≤ 0.05; **p ≤ 0.01; ***p ≤ 0.001).

### Supplementary information

Supplemental Material and Methods

Supplementary Figure Legends

Supplementary Figure 1: Establishment of mKATE2-NLS positive cell lines and growth conditions:

Supplementary Figure 2: Influence of Gln on metastatic features of mKATE2-NLS positive cell lines.

Supplementary Figure 3: Investigation of Gln deprivation on cellular functions.

Supplementary Figure 4: Isobologram analysis of combined treatment with Gln deprivation and docetaxel.

Supplementary Figure 5: Influence of PCa staging and treatment on GLS1 expression and overall survival.

Supplementary Figure 6: Influence of siGLS1 on GLS1 expression at different time points in PCa cell lines.

Supplementary Figure 7: Influence of CB-839 on metastatic features of mKATE2-NLS positive cell lines.

## Data Availability

Data sharing not applicable to this article as no datasets were generated or analysed during the current study. All data generated or analysed during this study are included in this published article and its supplementary information files.
