# Supplementary material for: Targeting the glutamine metabolism to suppress cell proliferation in mesenchymal docetaxel-resistant prostate cancer
Source: Oncogene. 2024 May 15;43(26):2038–50. doi: 10.1038/s41388-024-03059-4 (PMC11196217; doi:10.1038/s41388-024-03059-4)
Supplement: Supplementary file 1 — Supplemental Material and Methods [file 41388_2024_3059_MOESM1_ESM.docx]

Additional File 1: Supplementary Materials & Methods

mKATE2 nuclei labelling by lentiviral transduction

For cell counting using the IncuCyte^®^ S3 live-cell imaging system, all cell lines were stably transduced with mKATE2-NLS. For lentiviral particle production, 293T cells (ATCC) were transfected with psPAX2, pVSV-G, and the pLenti6.4-EF1a-mKate2-NLS expression vector at a ratio of 3:1:4 using ViaFect according to the manufacturer's instructions. psPAX2 was gifted by Didier Trono (Addgene plasmid # 12260; http://n2t.net/addgene:12260; RRID: Addgene_12260). The pVSV-G plasmid was a gift from Akitsu Hotta (Addgene plasmid # 138479; http://n2t.net/addgene:138479; RRID: Addgene_138479). pLenti6.4-EF1a-mKate2-NLS was provided by Dr. Handle (Medical University of Innsbruck, Innsbruck, Austria). Viral supernatants were harvested 48 h after transfection and passed through a FILTROPUR S 0.45 μm filter (Cat# 83.1826, Sarstedt, Nürnbrecht, Germany). Target cell lines were incubated for 8 h with the virus and 10 µg/ml polybrene Infection/Transfection Reagent and subsequently selected with 10 µg/mL blasticidin. To simplify readability, the mKATE2 label was removed.

Live cell count

For cell counting, the Muse Count & Viability Assay Kit (Cat# MCH100103, Luminex Corp, Austin, TX, USA) was used according to the manufacturer's instructions and analysed using a Muse^®^ Cell Analyzer (Luminex Corp).

IncuCyte® Cytotox assay

The IncuCyte^®^ live-cell analysis system was used to determine the cytotoxicity levels of the treated cells. After seeding the cells into a 96-well plate and incubating them for 24 h, the Cytotox reagent (Cat# 4633, Sartorius AG) was diluted in medium to 250 nM and added to the cells along with the treatment. The plates were placed in the IncuCyte^®^ S3 Live-Cell Analysis System (Sartorius AG), and the first scan was performed 30 min after treatment, repeated every 6 h. The data were analysed using the IncuCyte 2023C analysis software (Sartorius AG). The Cytotox signal was normalised to the cell number in the measured wells for statistical evaluation.

Clonogenic assay and Clonogenic recovery assay

For the clonogenic survival assay, 200 cells/well were seeded per well and treated with Gln or CB-839 for 10 days. Subsequently, the medium was removed, and the formed colonies were fixed with ice-cold methanol for 10 min and stained with 0.05% crystal violet solution (Sigma-Aldrich) for 30 min. The number and size of the cell clone colonies were determined using an Azure Sapphire Biomolecular Imager (Azure Biosystems, Biozym, Hess. Oldendorf, Germany) as reported before [1]. Colony-forming efficiency (CFE) was evaluated by ImageJ analysis, and the surviving fraction was calculated as reported before [2]. A colony was defined as containing at least 50 cells. The minimum size required was set individually for each cell line.

Migration and Invasion Assay

The migration experiments were performed as previously described [3]. For the migration and invasion experiments, 50,000 cells/well were seeded into IncuCyte^®^ Imagelock 96-well plates (Cat# BA-04856, Sartorius AG). For the invasion experiments, the plates were coated with 1% Matrigel^®^ matrix (Cat# 354230, Corning GmbH, Kaiserslautern, Germany) overnight at 37 °C. Subsequently, cells were seeded and incubated overnight (37 °C with 5% CO_2_). After 24 h, wounds were made using the Incucyte^®^ Cell Migration Kit (Cat# BA-04858, Sartorius AG). After two washing steps with a culture medium to remove the detached cells, the cells were treated for the experiments. The IncuCyte^®^ S3 Live-Cell Imaging System (Sartorius AG)was used to monitor wound density, and images were taken every 4 h for 48 h. The data were analysed using the IncuCyte 2023C analysis software (Sartorius AG).

Spheroid forming assay

Spheroid formation and growth capacity were assessed according to the IncuCyte^®^ Single Spheroid Assay protocol. To this end, 1,500 cells/well for PC3 cells and 6,000 cells/well for DU145 cells were seeded into 96-well BIOFLOAT^TM^ plates with U-bottom (Cat# F202003, faCellitate, Mannheim, Germany). After seeding and treatment, the plate was centrifuged (500 × g, 1 min, 20 °C), placed in the IncuCyte^®^ S3 Live-Cell Imaging System (Sartorius AG) and scanned every 6 h. Spheroid formation was evaluated for 96 h after treatment. After spheroid formation, the medium changes and spheroid growth were evaluated for 120 h. IncuCyte 2023C analysis software (Sartorius AG) was used for data analysis.

Analysis of reactive oxygen species (ROS)

Cells were seeded in 96-well plates (Cat# 3596, Corning GmbH) to investigate ROS induction and incubated under the respective treatments. Subsequently, the medium was discarded, and 10 µM of the ROS indicator 2',7'-dichlorodihydrofluorescein diacetate (H2DCFDA, Cat# D6883, Sigma-Aldrich) in PBS Buffer (Cat# D8537, Sigma-Aldrich) was added. 50 µM H_2_O_2_ (Cat# H1009, Sigma-Aldrich) was the positive control for ROS induction. After 15 min of incubation, the signal was detected using IncuCyte^®^ S3 Live Cell Imager (Sartorius AG). The data were analysed using IncuCyte 2023C analysis software. The ROS signal was normalised to the number of cells in the well.

Cell cycle analyses

The Click-iT™ Plus EdU 488 Flow Cytometry Assay Kit (Cat# C10632, Thermo Fisher Scientific) was used according to the manufacturer's instructions. Cells were measured using a MACSQuant Analyzer 10 flow cytometer (Miltenyi Biotec, Bergisch Gladbach, Germany) using 25,000 events in each sample and analysed using FlowJo software version 10.6.2 (FlowJo, Vancouver, BC, Canada).

siRNA-mediated gene expression knockdown

For glutaminase (GLS)1 knockdown, cells were transfected with Lipofectamine^®^ 2000 (Cat# 11668027, Thermo Fisher Scientific) according to the manufacturer's protocol. Eurofins synthesised the siRNAs and dissolved them in siRNA buffer (Cat# B-002000-UB-100, Dharmacon, Lafayette, USA) to a concentration of 10 µM. The siRNA sequences used are listed in Supplementary Table**S2.**

RNA Isolation and Quantitative Real-Time PCR

RNA isolation and quantitative real-time polymerase chain reaction were performed as described [4, 5]. The geometric means of *HPRT1* and *TBP* were used for normalisation. The LightCycler^®^ 480 software version 1.5 (Roche, Mannheim, Germany) was used to determine crossover point values. The ΔCp = Cp_GOI_-Cp_Housekeeper_ values were calculated and expressed as 2^−ΔCp^. The following primers were used: *GLS* (Hs01014020_m1), *HPRT1* (Hs02800695_m1), *TBP* (Hs00427620_m1).

Western blotting

Cell harvesting, protein determination, and western blotting were performed as described earlier [5]. Signals were acquired using a Microchemi chemiluminescence system (DNR Bio-Imaging Systems, Jerusalem, Israel). The uncropped western blot images are shown in the supplementary files. Densitometric analysis was performed using the Image-Studio Lite 5.2 software (LI-COR Biosciences, Lincoln, USA). Supplementary Table**S3** lists the antibodies used by the company and the dilutions applied.

Seahorse analysis

To assess mitochondrial function, a Seahorse XF Cell Mito Stress Test Kit (Cat# 103015-100, Agilent Technologies, Waldbronn, Germany) was used on an Agilent Seahorse XFp analyser (Agilent Technologies) following the manufacturer's instructions. 20,000 PC3 and DU145 cells were seeded in 180 μl medium in an Agilent miniplate (Agilent Technologies) the day before the assay and cultured overnight. The following day, the medium was changed to XF RPMI medium (pH 7.4; Agilent Technologies) and the cells were incubated in a non-CO2 incubator at 37 °C for 45 min. Inhibitors were diluted in assay medium and added to the sensor cartridge: Oligomycin (1.5 μM final concentration, Cat# Cay11342-5, Cayman Chemical Company, Ann Arbor, USA), FCCP (Cat# Cay15218-10, 1 μM final concentration, Cayman Chemical Company) and Rotenone/Antimycin A (1 μM final concentration, Cat# R8875/A8674, both Sigma Aldrich). After the assay, Calcein AM (1 μg/mL, Cat# ION-1071B, BIOZOL Diagnostica Vertrieb GmbH, Eching, Germany) was added to the wells for cell count normalisation. The plates were incubated for 10 min and imaged at 4x magnification using an EVOS M5000 fluorescence microscope (Thermo Fisher Scientific). Images were analysed using the FIJI software, and cell counts were used for normalisation. All metabolic data were analysed using built-in Agilent Seahorse Wave software (Agilent Technologies).

**Supplementary Table S1: Cell line characteristics**

| Cell line name | Characteristics | Origin | Media | Reference |
| --- | --- | --- | --- | --- |
| PC3 CTRL | - Small cell carcinoma - Androgen independent - Docetaxel sensitive - Epithelial Phenotype - mKATE2-NLS positive | Bone metastasis | RPMI-1640 | (1-3) |
| PC3 DR | - Androgen independent - Docetaxel resistant - Mesenchymal Phenotype - mKATE2-NLS positive | Bone metastasis | RPMI-1640  + 10 nm DX | (1, 4) |
| DU145 CTRL | - Androgen independent - Epithelial Phenotype - Docetaxel sensitive - mKATE2-NLS positive | Brain metastasis | RPMI-1640 | (1, 5) |
| DU145 DR | - Androgen independent - Docetaxel resistant - Mesenchymal Phenotype - mKATE2-NLS positive | Brain metastasis | RPMI-1640  + 10 nm DX | (1, 4) |

**Supplementary Table S2: siRNA Sequences as previously published by Muhka et al. (6)**

| **Name** | **Target sequence (5'-3')** |
| --- | --- |
| siCTRL | 5’-GGCUAAAGGAAACGAAAGA-3’ |
| siGLS1#1 | 5’-GCAGUUCGAAAUACAUUGA-3’ |
| siGLS1#2 | 5’-GGGUCUGUUACCUAGCUUG-3’ |
| siGLS1#3 | 5’-GGACAAGAGAAAAUACCUG-3’ |

**Supplementary Table S3: Antibodies used in this study for western blot**

| **Name** | **Lot.** | **Cat#** | **Origin** | **Company** | **Dillution** |
| --- | --- | --- | --- | --- | --- |
| Glutaminase-1/GLS1 (E9H6H) XP^R^ Rabbit mAb | 1 | #88964 | rabbit | Cell Signaling Technology | 1:10.000 |
| Cleaved PARP (Asp214) Antibody (Human Specific) | 15 | #9541 | rabbit | Cell Signaling Technology | 1:2000 |
| Mouse Monoclonal anti-GAPDH (6C5cc) | 19/05-G4cc-C5cc | NB600-502 | mouse | Novus Biologicals | 1:10.000 |
| Polyclonal Swine Anti-Rabbit HRP | 00059646 | P039901-2 | mouse | Agilent Technologies | 1:10.000 |
| Polyclonal Rabbit Anti-Mouse HRP | 20066043 | P026002-2 | mouse | Agilent Technologies | 1:10.000 |

**Supplemetary References**

1 Markowitsch SD, Schupp P, Lauckner J, Vakhrusheva O, Slade KS, Mager R *et al*. Artesunate Inhibits Growth of Sunitinib-Resistant Renal Cell Carcinoma Cells through Cell Cycle Arrest and Induction of Ferroptosis. *Cancers* 2020; doi:10.3390/cancers12113150.

2 Nappo G, Handle F, Santer FR, McNeill RV, Seed RI, Collins AT *et al*. The immunosuppressive cytokine interleukin-4 increases the clonogenic potential of prostate stem-like cells by activation of STAT6 signalling. *Oncogenesis* 2017; 6: e342.

3 Erb HHH, Ebert M, Kuhn R, Donix L, Haferkamp A, Seed RI *et al*. PIAS1 is not suitable as a urothelial carcinoma biomarker protein and pharmacological target. *PloS one* 2019; 14: e0224085.

4 Sommer U, Siciliano T, Ebersbach C, Beier AK, Stope MB, Jöhrens K *et al*. Impact of Androgen Receptor Activity on Prostate-Specific Membrane Antigen Expression in Prostate Cancer Cells. *Int J Mol Sci* 2022; 23.

5 Erb HH, Bodenbender J, Handle F, Diehl T, Donix L, Tsaur I *et al*. Assessment of STAT5 as a potential therapy target in enzalutamide-resistant prostate cancer. *PloS one* 2020; 15: e0237248.
