# Supplementary material for: Targeting the glutamine metabolism to suppress cell proliferation in mesenchymal docetaxel-resistant prostate cancer
Source: Oncogene. 2024 May 15;43(26):2038–50. doi: 10.1038/s41388-024-03059-4 (PMC11196217; doi:10.1038/s41388-024-03059-4)
Supplement: Supplementary file 2 — Supplementary Figure Legends [file 41388_2024_3059_MOESM2_ESM.docx]

Supplementary Figure Legends:

**Supplementary Figure 1**: **Establishment of mKATE2-NLS positive cell lines and growth conditions:** (A) Analysis of mKATE2 positive cells after blasticidin selection. mKATE2-NLS positive cells were detected using the Keyence microscope and analysed with the BZ-X800 Analyzer software. The graphical illustration of the mKATE2-NLS positive cells for each cell line was plotted as box and whisker (min to max). (B) Correlation analysis of mKATE2 positive and negative cell confluence using the S3 Incucyte® Live-Cell Analysis System. Pearson correlation (r) was calculated using the Prism software. (C) Dose-response curves of different concentrations of docetaxel and graphical illustration of the change in IC_50_ values of PC3-CTRL, PC3-DR, DU145-CTRL, and DU145-DR cell proliferation compared to 2 mM Gln. Data was plotted as mean±SEM of the three biological replicates. Significant differences were identified using paired Student T-Test. (D) Relative changes in intrinsic growth rates of DU145-CTRL and DU145-DR cells cultured with different concentrations of FBS in the presence and absence of 2 mM Gln. Data was plotted as mean±SEM of the three biological replicates. Significant differences were identified using One-way ANOVA. (E) Relative changes in intrinsic growth rates of PC3-CTRL, PC3-DR, DU145-CTRL, and DU145-DR cells cultured with different concentrations of FBS. Data was plotted as mean±SEM of the three biological replicates. Significant differences were identified using One-way ANOVA. All differences highlighted by asterisks were statistically significant (*: p ≤ 0.05. **: p ≤ 0.01 *** p ≤ 0.001).

**Supplementary Figure 2: Influence of Gln on metastatic features of mKATE2-NLS positive cell lines.** (A) Quantification of the adhesion capacity of PC3-CTRL, PC3-DR, DU145-CTRL, and DU145-DR cells after 96 h of Gln deprivation to normal associated fibroblasts (NAF), cancer-associated fibroblasts (CAF), Human umbilical vein endothelial cells (HUVEC), Matrigel^®^, or Poly-D-Lysine. Data represent mean±SEM of three independent experiments. (B) Clonogenic assays of PC3-CTRL, PC3-DR, DU145-CTRL, and DU145-DR cells after Gln withdrawal. Survival Fraction represents the relative change in colony number (⩾50 cells/colony). Average colony size represents the relative change in colony area. Data is shown as relative changes compared to 2 mM Gln and was scored 10 days after plating. The results are plotted mean±SEM of four biological replicates. (C) Statistical analysis of the wound heal assays in PC3-CTRL, PC3-DR, DU145-CTRL, and DU145-DR cells after Gln deprivation. Data are expressed as relative change of wound width in μm and wound confluence in % and are the mean±SEM of three independent experiments. (D) Statistical analysis of the invasion assays in PC3-CTRL, PC3-DR, DU145-CTRL, and DU145-DR cells after Gln deprivation. Data are expressed as relative change of wound width in μm and wound confluence in % and are the mean±SEM of three independent experiments.

**Supplementary Figure 3: Investigation of Gln deprivation on cellular functions.** (A) Influence of Gln deprivation combined with glutathione treatment on PC3-CTRL, PC3-DR, DU145-CTRL, and DU145-DR cell proliferation for 96 h. Curve fitting was performed using Prism. Values are expressed as mean±SEM relative to 0 h. (B) Seahorse Cell Mito Stress analysis of PC3-CTRL, PC3-DR, DU145-CTRL, and DU145-DR after 24 h Gln starvation. Data are normalised to cell number and represent the mean±SEM of three independent experiments. (C) Relative change of cell cycle phases after 96 h Gln deprivation compared to 2 mM Gln. Data is represented as the mean±SD of three independent experiments. (D) Influence of Gln rechallenge after 120 h Gln deprivation on DU145-CTRL and DU145-DR cell proliferation. Curve fitting was performed using Prism. Values are expressed as mean±SEM relative to 0 h. (E) Uncropped western blot images of cPARP and GAPDH.

**Supplementary Figure 4: Isobologram analysis of combined treatment with Gln deprivation and docetaxel.** (A) Results of dose-response experiments of PC3-CTRL and DU145-CTRL after 96 h of Gln deprivation and docetaxel treatment. Data is shown as mean±SEM. of three independent experiments. (B) Graphical illustration of the combination index (CI) in PC3-CTRL and DU145-CTRL. (C) Results of dose-response experiments of PC3-DR and DU145-DR after 96 h of Gln deprivation and docetaxel treatment. Data is shown as mean±SEM of three independent experiments. (D) Immunohistochemical analysis of paraffin-embedded human colon carcinoma using Glutaminase-1/GLS1 (E9H6H) XP® Rabbit mAb (left) compared to concentration-matched isotype control. (E) GLS1 antibody specificity for immunohistochemical experiments was controlled using formalin-fixed paraffin-embedded untransfected PC3 or PC3 transfected with siGLS1 #1. Intensity evaluation and nuclei counting were performed using Fiji, as in Eigentler et al. (60, 61).

**Supplementary Figure 5: Influence of PCa staging and treatment on GLS1 expression and overall survival.** (A) Influence of Gleason score and TMN staging on GLS1 expression of the Innsbruck cohort. (B) Analysis of GLS1 expression of the docetaxel-treated patients of the Dresden cohort. (C) Kaplan–Meier curves indicating OS according to the GLS1 expression level in the docetaxel-treated patients of the Dresden cohort. (E) Uncropped western blot images of GLS1 and GAPDH.

**Supplementary Figure 6: Influence of siGLS1 on GLS1 expression at different time points in PCa cell lines.** (A) Uncropped western blot images of GLS1 and GAPDH after 24 h of siRNA transfection. (B) Uncropped western blot images of GLS1 and GAPDH after 48 h of siRNA transfection. (C) Densitometric analysis for changes in GLS1 protein levels after 24 h and 48 h siRNA transfection in PC3-CTRL, PC3-DR, DU145-CTRL, and DU145-DR cells normalised to GAPDH. Values are expressed mean±SD. Significant differences were identified using one-way ANOVA. (D) Uncropped western blot images of GLS1 and GAPDH after 96 h of siRNA transfection. All differences highlighted by asterisks are statistically significant (*: p ≤ 0.05. **: p ≤ 0.01 *** p ≤ 0.001).

**Supplementary Figure 7: Influence of CB-839 on metastatic features of mKATE2-NLS positive cell lines.** (A) Clonogenic assays of PC3-CTRL, PC3-DR, DU145-CTRL, and DU145-DR cells after CB-839 treatment. Survival Fraction represents the relative change in colony number (⩾50 cells/colony). Average colony size represents the relative change in colony area. Data is shown as relative changes compared to 2 mM Gln and was scored 10 days after plating. The results are plotted mean±SEM of four biological replicates. (B) Statistical analysis of the wound heal assays in PC3-CTRL, PC3-DR, DU145-CTRL, and DU145-DR cells after Gln deprivation or 1 µM CB-839 treatment. Data are expressed as relative change of wound width in μm and wound confluence in % and are the mean±SEM of three independent experiments. (C) Statistical analysis of the invasion assays in PC3-CTRL, PC3-DR, DU145-CTRL, and DU145-DR cells after Gln deprivation or 1 µM CB-839 treatment. Data are expressed as relative change of wound width in μm and wound confluence in % and are the mean±SEM of three independent experiments. (D) Seahorse Cell Mito Stress analysis of PC3-CTRL, PC3-DR, DU145-CTRL, and DU145-DR after 24 h CB-839 (1 µM) treatment. Data are normalised to cell number and represent the mean±SEM of three independent experiments. (E) Relative mitochondrial respiration changes with basal respiration, proton leak, maximal respiration, non-mitochondrial oxygen consumption, and ATP production. Data was obtained by performing a Seahorse Cell Mito Stress analysis. The results are plotted mean±SEM of three biological replicates. Significant differences were identified using one-way ANOVA. (F) Results of dose-response experiments and changes in IC_50_ values of PC3-DR and DU145-DR after 96 h of CB-839 and 50 nM Elacridar treatment. Data is shown as mean±SEM of three independent experiments.
