# Supplementary figures and images for: Targeting the glutamine metabolism to suppress cell proliferation in mesenchymal docetaxel-resistant prostate cancer
Source: Oncogene. 2024 May 15;43(26):2038–50. doi: 10.1038/s41388-024-03059-4 (PMC11196217; doi:10.1038/s41388-024-03059-4)

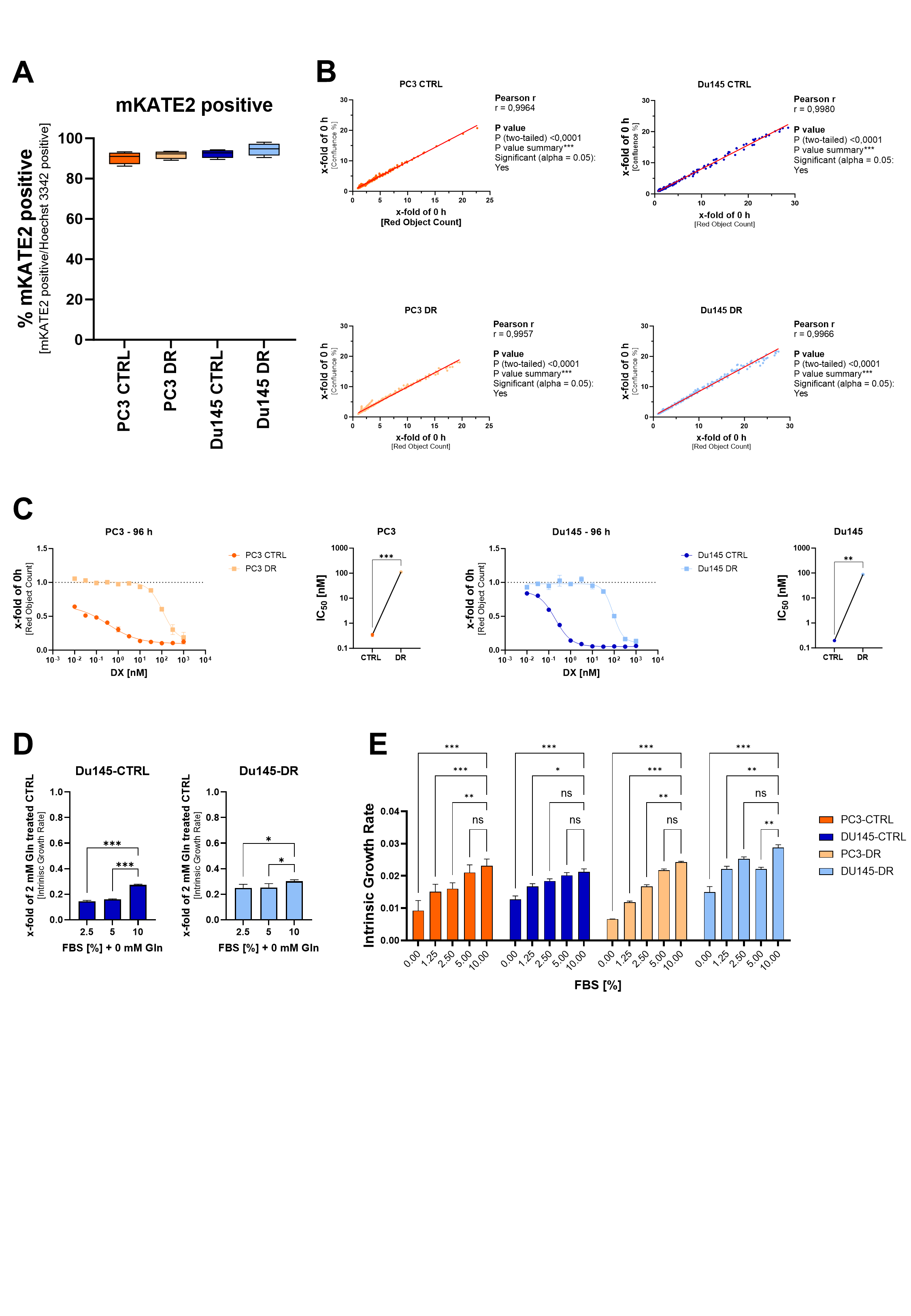

Supplement: Supplementary file 3 — Supplementary Figure 1: Establishment of mKATE2-NLS positive cell lines and growth conditions: [file 41388_2024_3059_MOESM3_ESM.tif]

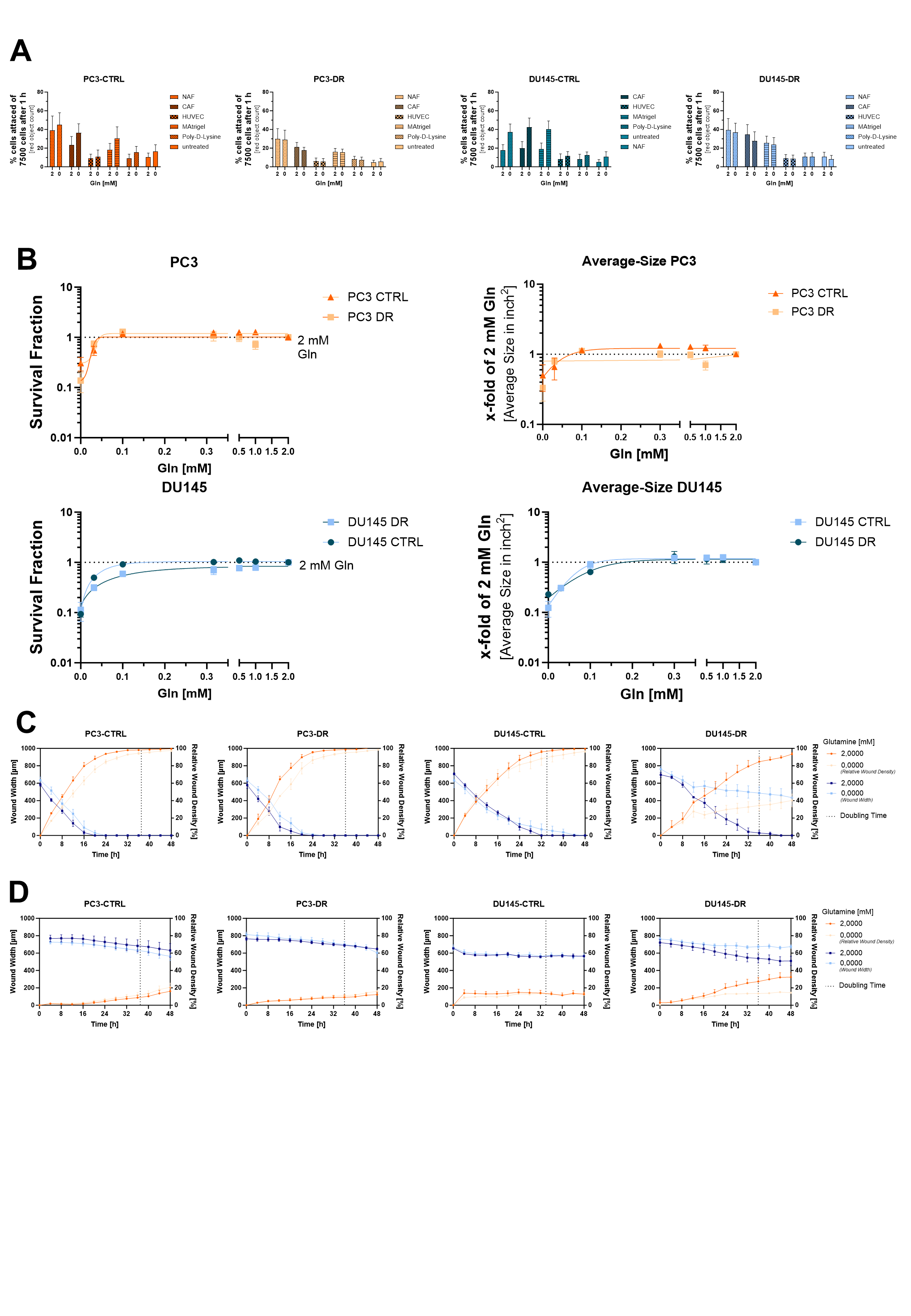

Supplement: Supplementary file 4 — Supplementary Figure 2: Influence of Gln on metastatic features of mKATE2-NLS positive cell lines. [file 41388_2024_3059_MOESM4_ESM.tif]

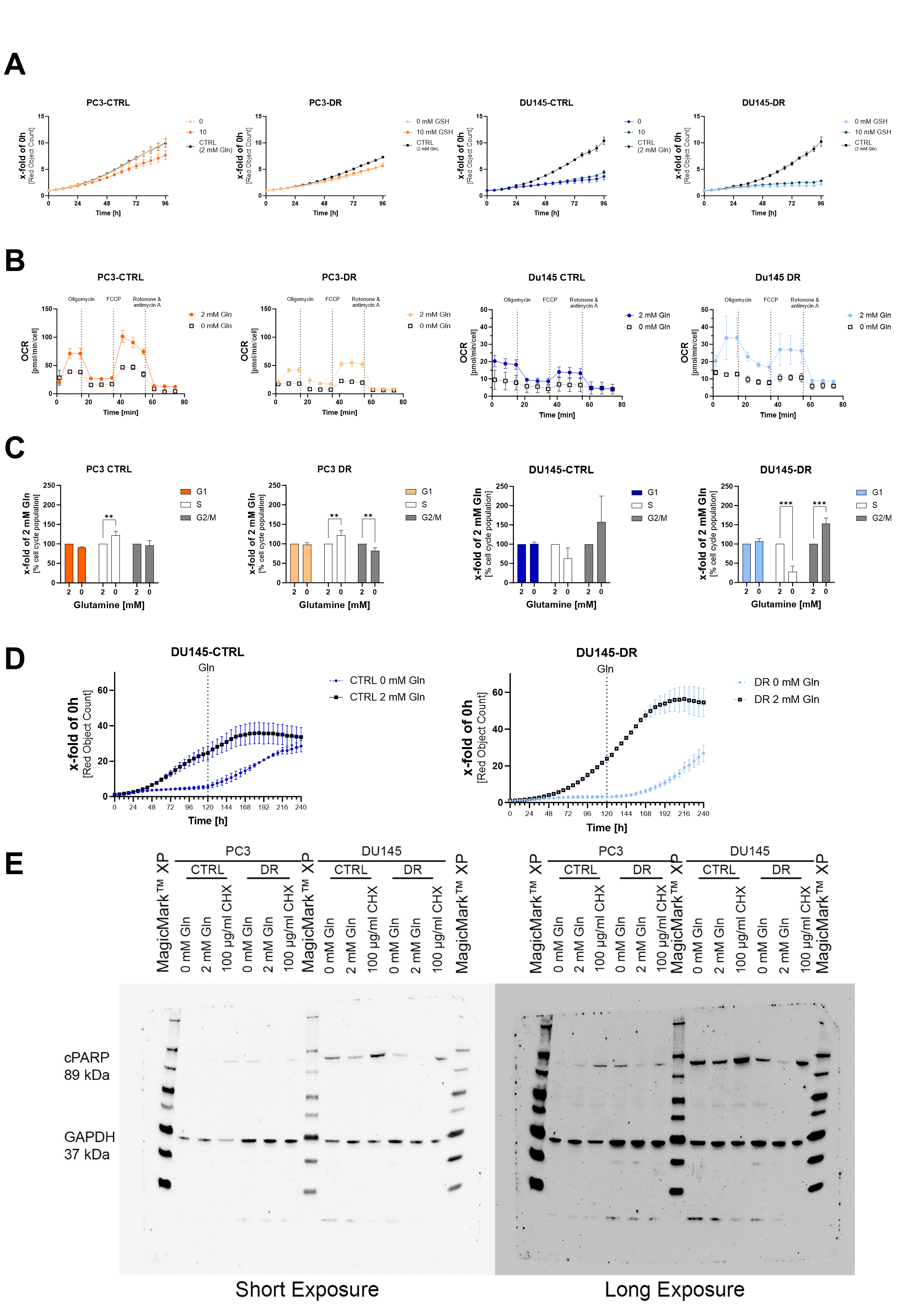

Supplement: Supplementary file 5 — Supplementary Figure 3: Investigation of Gln deprivation on cellular functions. [file 41388_2024_3059_MOESM5_ESM.tif]

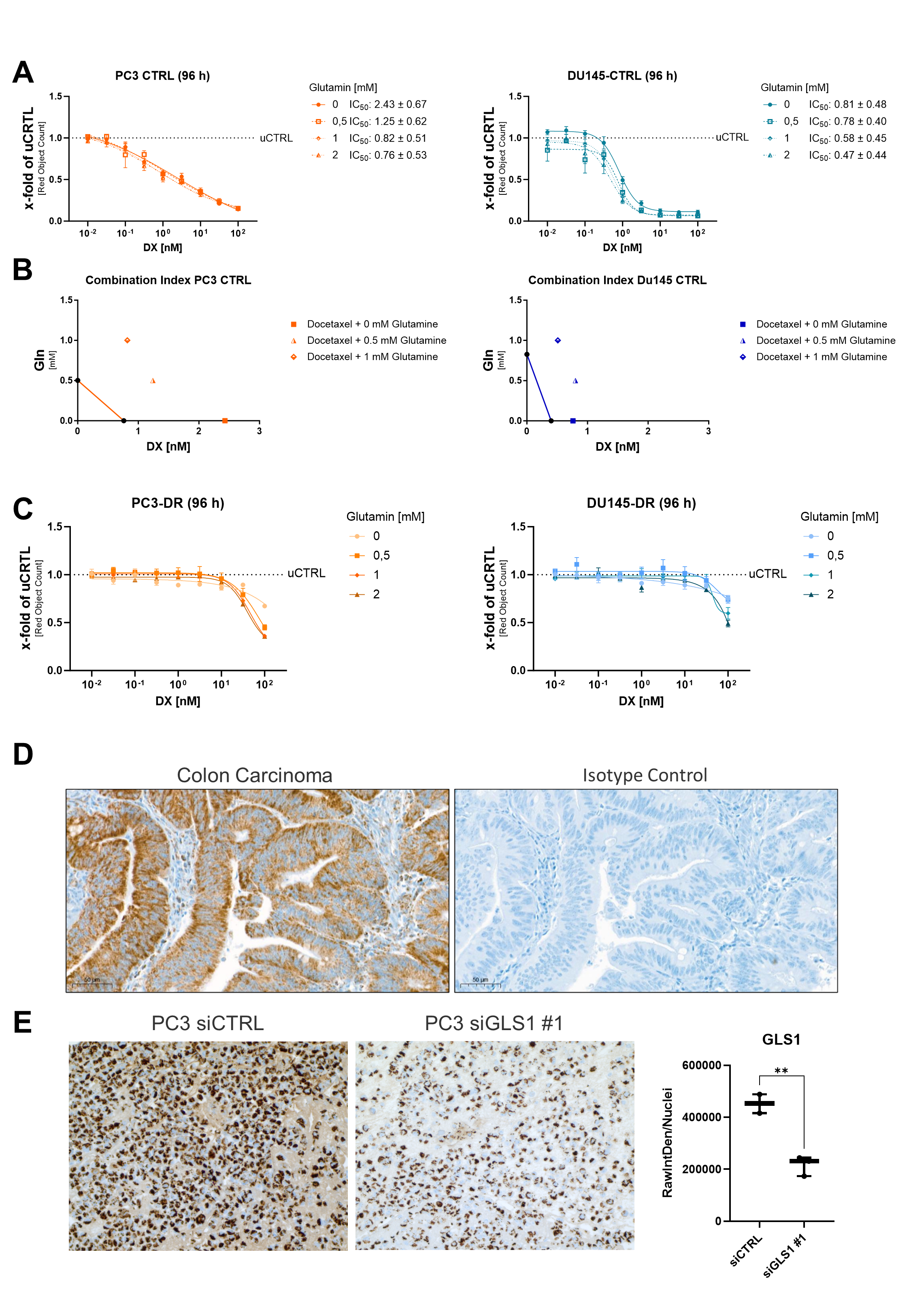

Supplement: Supplementary file 6 — Supplementary Figure 4: Isobologram analysis of combined treatment with Gln deprivation and docetaxel. [file 41388_2024_3059_MOESM6_ESM.tif]

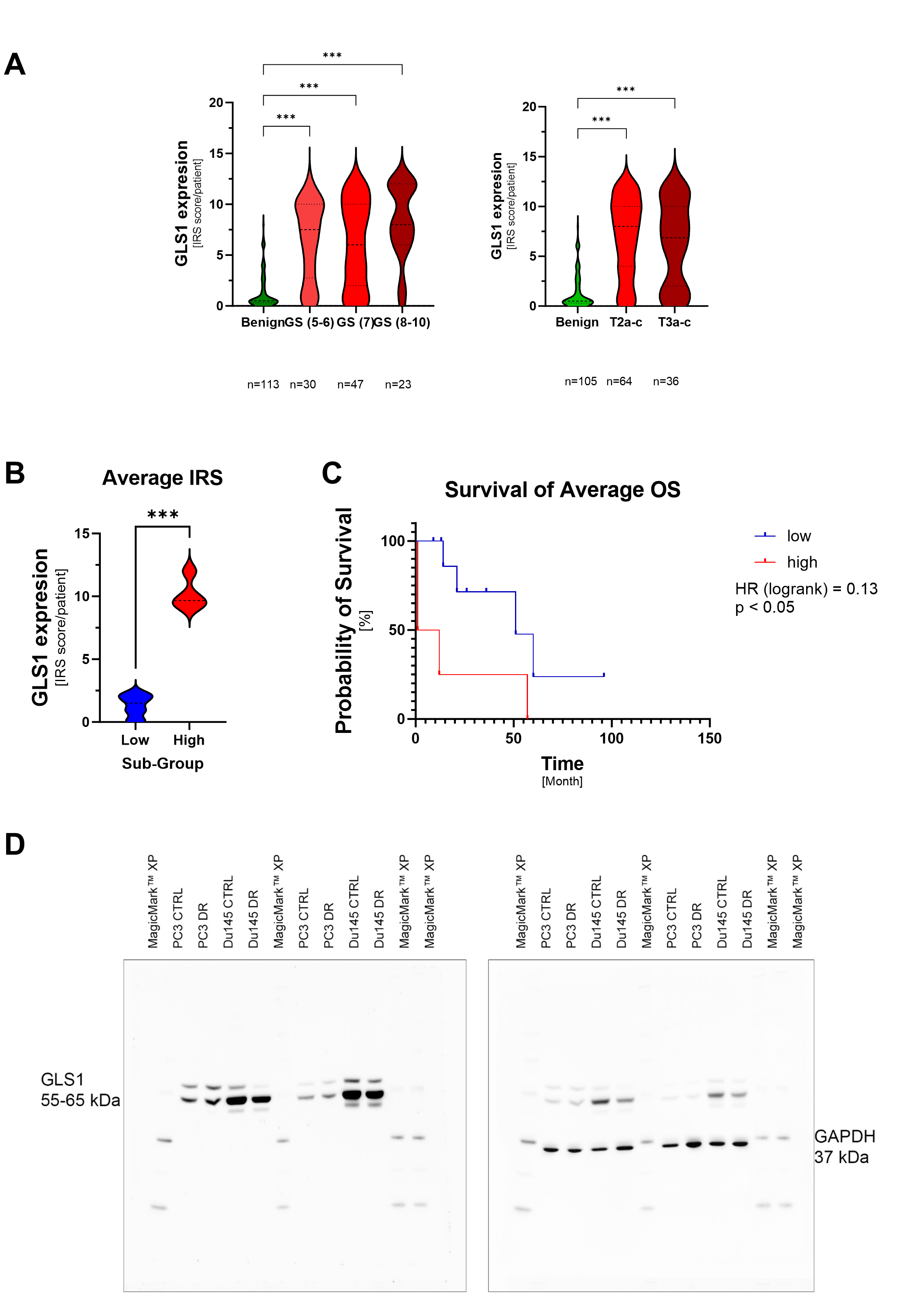

Supplement: Supplementary file 7 — Supplementary Figure 5: Influence of PCa staging and treatment on GLS1 expression and overall survival. [file 41388_2024_3059_MOESM7_ESM.tif]

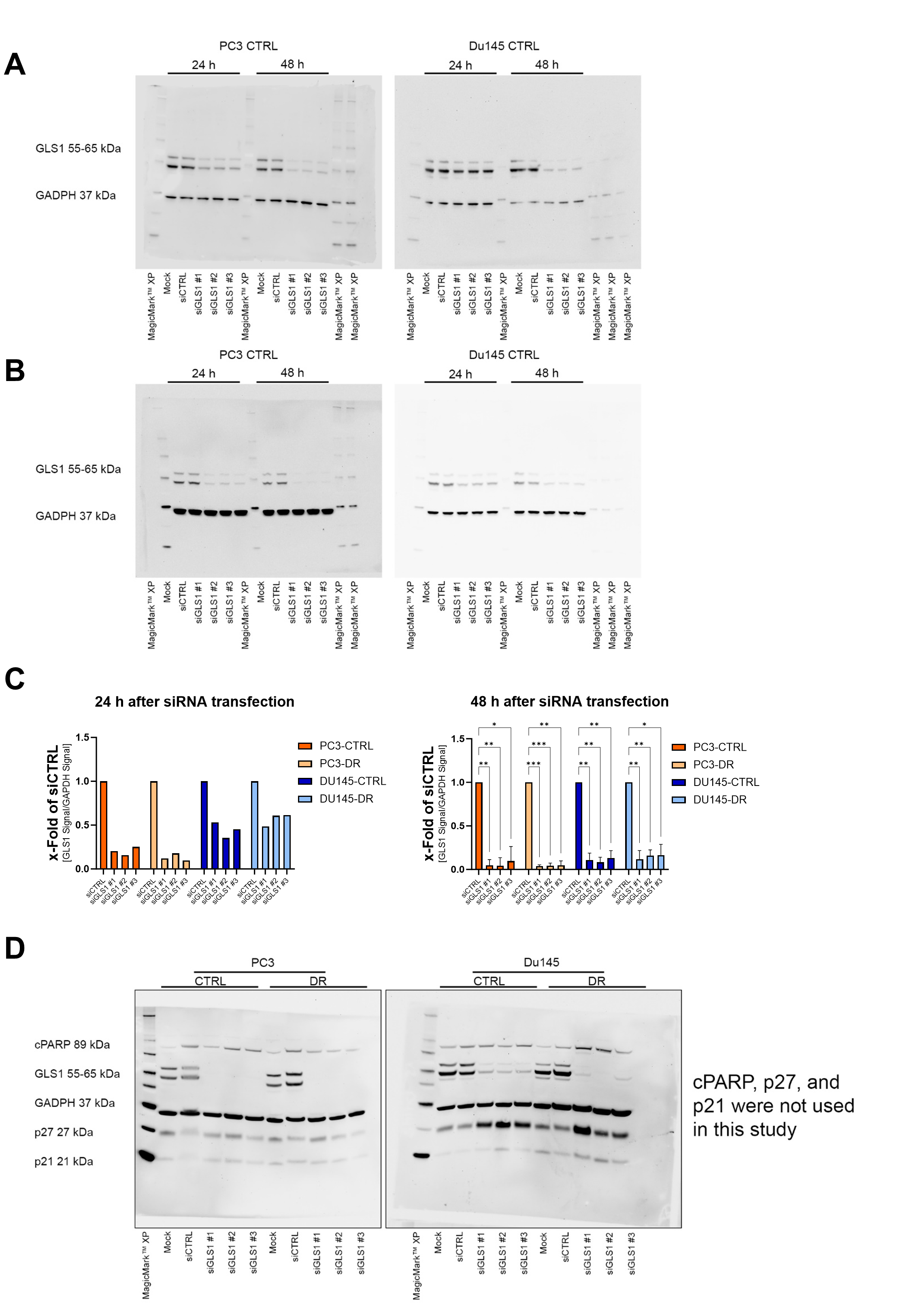

Supplement: Supplementary file 8 — Supplementary Figure 6: Influence of siGLS1 on GLS1 expression at different time points in PCa cell lines. [file 41388_2024_3059_MOESM8_ESM.tif]

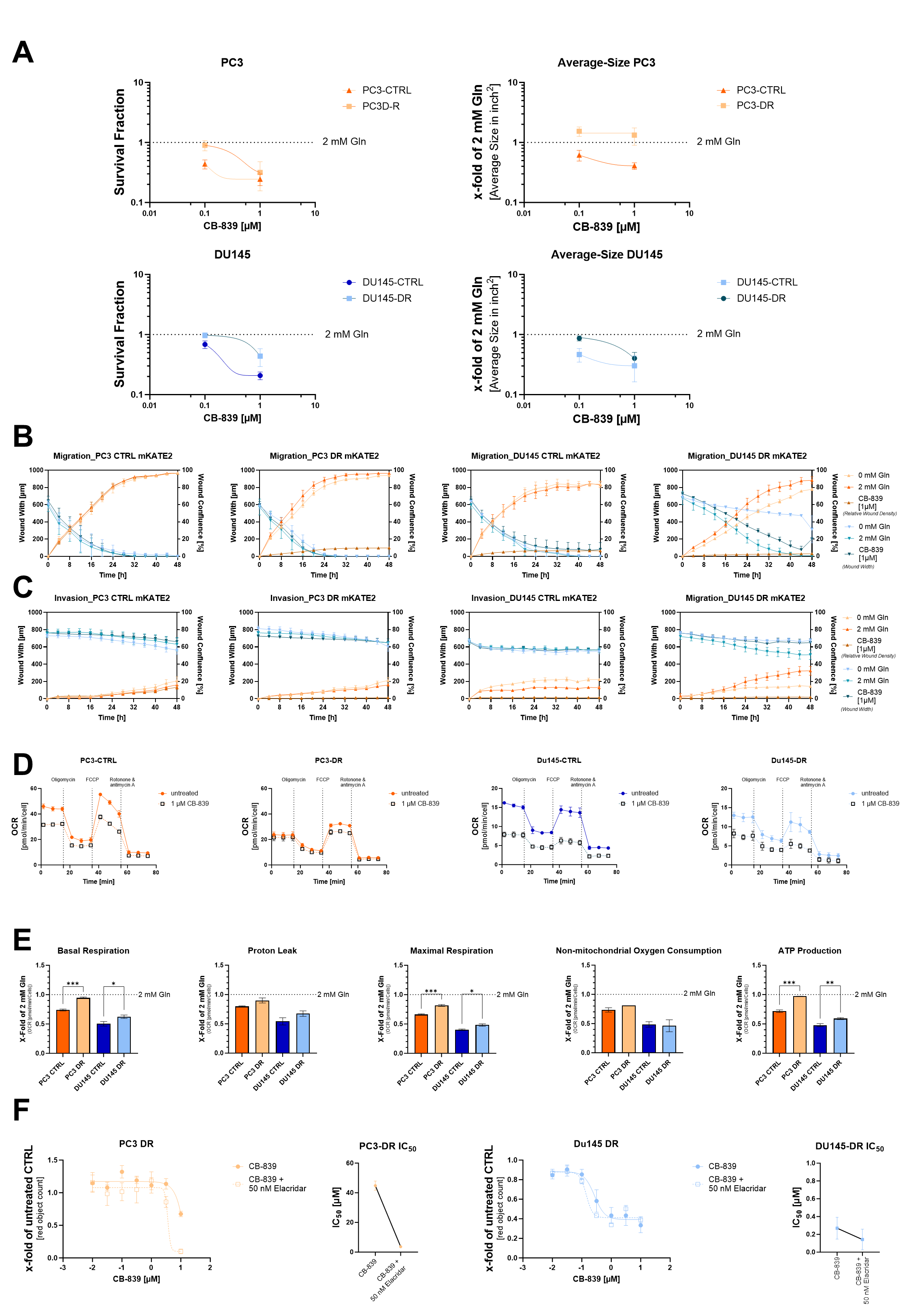

Supplement: Supplementary file 9 — Supplementary Figure 7: Influence of CB-839 on metastatic features of mKATE2-NLS positive cell lines. [file 41388_2024_3059_MOESM9_ESM.tif]
